# The more the better: on the formation of single-phase high entropy alloy nanoparticles as catalysts for the oxygen reduction reaction†

**DOI:** 10.1039/d3ey00201b

**Published:** 2023-08-22

**Authors:** Rebecca K. Pittkowski, Christian M. Clausen, Qinyi Chen, Dragos Stoian, Wouter van Beek, Jan Bucher, Rahel L. Welten, Nicolas Schlegel, Jette K. Mathiesen, Tobias M. Nielsen, Jia Du, Asger W. Rosenkranz, Espen D. Bøjesen, Jan Rossmeisl, Kirsten M. Ø. Jensen, Matthias Arenz

**Affiliations:** a Center for High Entropy Alloy Catalysis (CHEAC), Department of Chemistry, University of Copenhagen Copenhagen Denmark Rebecca.pittkowski@chem.ku.dk kirsten@chem.ku.dk; b Swiss Norwegian Beamline, European Synchrotron Radiation Facility (ESRF) Grenoble France; c Department of Chemistry, Biochemistry and Pharmaceutical Sciences, University of Bern Bern Switzerland matthias.arenz@unibe.ch; d Department of Physics, Technical University of Denmark Kgs. Lyngby Denmark; e Aarhus University, Interdisciplinary Nanoscience Center Aarhus Denmark

## Abstract

High entropy alloys (HEAs) are an important new material class with significant application potential in catalysis and electrocatalysis. The entropy-driven formation of HEA materials requires high temperatures and controlled cooling rates. However, catalysts in general also require highly dispersed materials, *i.e.*, nanoparticles. Only then a favorable utilization of the expensive raw materials can be achieved. Several recently reported HEA nanoparticle synthesis strategies, therefore, avoid the high-temperature regime to prevent particle growth. In our work, we investigate a system of five noble metal single-source precursors with superior catalytic activity for the oxygen reduction reaction. Combining *in situ* X-ray powder diffraction with multi-edge X-ray absorption spectroscopy, we address the fundamental question of how single-phase HEA nanoparticles can form at low temperatures. It is demonstrated that the formation of HEA nanoparticles is governed by stochastic principles and the inhibition of precursor mobility during the formation process favors the formation of a single phase. The proposed formation principle is supported by simulations of the nanoparticle formation in a randomized process, rationalizing the experimentally found differences between two-element and multi-element metal precursor mixtures.

## Abstract 2

With *in situ* X-ray powder diffraction and multi-edge X-ray absorption spectroscopy, we probe high entropy alloy nanoparticle formation. When inhibiting precursor mobility, single-phase formation is governed by stochastics and favored for five elements.

## Graphical abstract

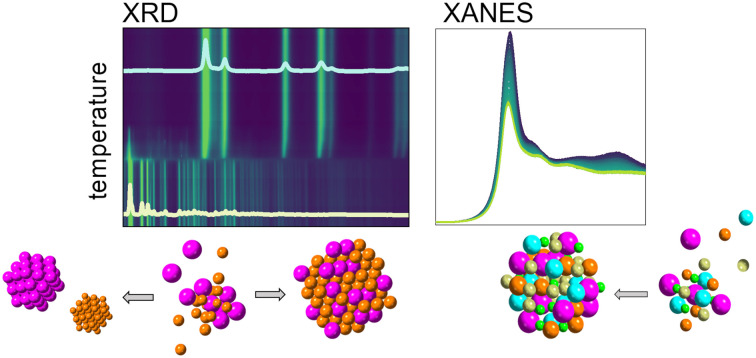

## 1. Introduction

High entropy alloys (HEAs), *i.e.* multi-metallic alloys of at least five elements,^1^ have been studied as a new material class with exceptional mechanical properties since 2004.^2–8^ More recently, HEAs have come into focus as a promising catalyst concept.^9–14^ By random mixing of multiple elements within a material, an almost unlimited number of variable surface atom arrangements can be achieved.^9^ This allows tuning the catalyst's surface and unlocks a statistical approach to catalyst design. For example, experimental investigations showed that indeed PdCuPtNiCo HEAs/C exhibit superior activity for the oxygen reduction reaction (ORR).^15^ However, typically such experimental studies comprise a rather small and random selection of different HEA compositions and are mostly descriptive. Combined experimental and theoretical studies that utilize the predictions from computational screenings are still scarce and mainly focus on thin film catalyst materials prepared by magnetron sputtering that can be more easily performed in experimental high throughput screening studies.^16,17^ To be able to predict and simulate the variety of binding sites of the different elements present in a HEA, single-phase materials are crucial.^10^ If phase segregation occurs, the surface atoms are not randomly distributed anymore and the key assumption of the computational modeling is not valid. Thus the experimental and computational trends will diverge.^18^

HEAs are conventionally prepared as bulk materials employing high-temperature methods such as arc melting.^1,8^ Bulk materials have very low dispersion, *i.e.*, a low ratio of surface to bulk atoms. A large surface-to-bulk atom ratio is, however, vital for efficient catalysts. Nanoparticles possess such high dispersion. This generates a large interest in developing HEA nanoparticle synthesis methods. Many synthesis methods for HEA nanoparticles are high-energy shock methods such as Joule-heating,^19–21^ microwave heating,^22^ laser-ablation,^23^ and fast-bed pyrolysis.^24^ Recent studies propose that it is also possible to use low-temperature solution-based techniques to synthesize HEA nanoparticles^15,25–27^ or low-temperature reduction of solid precursors.^28,29^ Such methods open for fine-tuning both the composition and nanostructure of nanoparticles. For high-temperature synthesis approaches, entropy plays a role in stabilizing the multi-metallic phases, so that single-phase materials can be prepared.^30–32^ Thermal shock methods are also believed to freeze the material in a state of high entropy during controlled cool-down.^19,24^ For the solution-based and low-temperature reduction syntheses of HEA nanoparticles, the factors determining phase formation of HEAs are so far unclear, as the entropy term is rather small at synthesis temperatures far out of thermodynamic equilibrium.^25,28^ To study which drivers favor the formation of single-phase nanoparticles, in this work, we investigate the phase-formation mechanism of noble metal PtIrOsRhRu nanoparticles at low temperatures. During the reduction of the precursors, we can follow the particle formation in detail *in situ*.^33^ With simultaneous *in situ* X-ray powder diffraction (XRD) and five-element X-ray absorption spectroscopy (XAS), the reduction of all individual elements (Pt, Ir, Os, Rh, Ru) is followed alongside the crystallization of HEA nanoparticles. Comparing multimetallic precursor mixtures with bimetallic ones, we propose a model for the formation of single-phase nanoparticles. The essential conclusion of the model, *i.e.*, that combining multiple elements favors the formation of single-phase nanoparticles, will aid the design of future studies combining experimental investigations with computational simulations.

## 2. Materials and methods

### 2.1. Material synthesis

All samples were prepared following a literature procedure described by Yusenko *et al.*^29^ All compounds were prepared from commercial metal precursors (Sigma Aldrich). As cationic precursors the chloropentaammine metal chlorides of Ir, Rh, and Ru were used; Ir(NH_3_)_5_Cl]Cl_2_, [Rh(NH_3_)_5_Cl]Cl_2_, and [Ru(NH_3_)_5_Cl]Cl_2_. The anionic precursors were the ammonium hexachlorometallates of Ir, Pt, and Os; (NH_4_)_2_[IrCl_6_], (NH_4_)_2_[PtCl_6_], and (NH_4_)_2_[OsCl_6_]. In short, separate aqueous solutions of anionic metal precursors (hexachlorates) and cationic metal precursors (chloropentaamines) were prepared by dissolving the respective amounts of the individual metal salts in hot MilliQ water (90 °C). The amounts of the respective metal precursor salts used were calculated to obtain a final product of 50 mg of reduced metal. Concentrations of the metal solutions were calculated to obtain a total of 0.005 mol L^−1^, *i.e.* 2.5 mM cationic and 2.5 mM anionic solutions were prepared. For the Ru–Ir bimetallic sample this amounts, *e.g.* to a 2.5 mM solution of [Ru(NH_3_)_5_Cl]Cl_2_ and a 2.5 mM solution of (NH_4_)_2_[IrCl_6_]. The fcc-HEA sample was prepared to include a total molar fraction of 20% hcp-forming metals (10% of both Ru and Os, respectively) and 80% fcc-forming metals (26.7% of Rh, Ir, and Pt, respectively). For the hcp-HEA sample, the initial molar amounts were adjusted to 50% hcp-forming metals (25% of both Ru and Os, respectively) and 50% fcc-forming metals (16.7% of Rh, Ir, and Pt, respectively). The anionic and cationic precursor solutions were stirred under continuous heating for 30 minutes to ensure the complete mixing of the solutions. The hot anionic and cationic solutions were combined and upon mixing, the single-source precursor precipitated immediately as a powder. The solutions were left to cool and the crystalline precipitate was collected, washed with MilliQ water, and dried in air.

For *ex situ* characterization, alloy samples were prepared by thermal decomposition of the prepared crystalline single source-precursors in a reducing gas atmosphere (Carbagas, 5% H_2_ in Ar). The samples were calcined in a quartz-tube furnace (Gero SR-A 40-250/11) in quartz crucibles at 700 °C for 60 minutes (heating ramp 50 K min^−1^). The crystalline phases of the prepared samples were determined from powder X-ray diffraction (XRD) data using a STOE StadiP with a Cu Kα X-ray source in transmission geometry. The elemental composition was analyzed by energy-dispersive X-ray spectroscopy (EDX) using a Zeiss Gemini 450 scanning electron microscope equipped with an Oxford Instruments UltimMax 65 EDX detector.

Synchrotron X-ray powder diffraction and X-ray total scattering data for pair distribution function (PDF) analysis of the prepared high entropy alloy samples was collected at DESY P21.1 at PETRAIII; DESY in Hamburg, Germany, with a wavelength of 0.12224 Å. All *ex situ* samples were prepared as powders in Kapton capillaries and data was collected at room temperature under a helium atmosphere to limit the background contribution arising from air scattering.

### 2.2. Transmission electron microscopy (TEM) and scanning transmission electron microscopy – energy dispersive X-ray spectroscopy (STEM-EDX)

The prepared nanoparticles were washed in ethanol five times and centrifuged at 9000 rpm for 10 minutes. After washing and drying overnight, the nanoparticles were resuspended in ethanol and subsequently drop-casted onto Au 300 mesh grids with 2 nm carbon (Quantifoil®).

High-resolution transmission electron microscopy (HR-TEM) images were collected using a Talos FX-200 equipped with a Ceta 16M camera with samples mounted in a single tilt holder. To reduce the effect of aberrations a 100 mm objective aperture was used during imaging. STEM images were collected on the same microscope. A 10.5 mrad converged electron probe was used. The collection angle of the STEM high-angle annular darkfield (HAADF) images was 60–200 mrad. STEM-EDX datasets were collected using the ChemiSTEM system installed on the Microscope. The STEM-EDX data was binned (6 × 6) and background subtraction *via* linear interpolation around the relevant elemental peaks was performed before plotting the maps reflecting the spatial distribution of the different elements using the HyperSpy^34^ python library.

### 2.3. Density functional theory (DFT) calculations

To obtain a qualitative measure of the mixing enthalpies of the bimetallic and HEA systems in both fcc and hcp crystal structures, the total energy of several bulk structures was calculated with density functional theory. The calculations used the revised Perdew–Burke–Ernzerhof (RPBE)^35^ exchange–correlation functional as implemented in the GPAW^36,37^ code. Each system was calculated as both fcc and hcp structures containing 32 and 16 atoms per cell, respectively. The elements of each atom in the binary systems were chosen randomly with equal probability and the elements in the HEA systems were chosen with probabilities corresponding to the precursor compositions: Ru_0.11_Rh_0.26_Os_0.06_Ir_0.22_Pt_0.35_ for the fcc-structure and Ru_0.31_Rh_0.09_Os_0.30_Ir_0.15_Pt_0.15_ for the hcp-structure. In all cases, the cell was scaled to the weighted mean of the lattice constants of the atoms constituting the cell. The wave functions were expanded in plane waves with an energy cut-off set to 600 eV, and the Brillouin zone was sampled with a Monkhorst–Pack grid^35^ of 4  ×  4  ×  4 and 8 × 8 × 4 *k*-points, for fcc and hcp structures respectively. 50 structures of each binary system were optimized until the maximum force on any atom was below at least 0.1 eV Å^−1^. For the HEA systems, 500 structures were calculated.

Subsequently, the mixing contribution was calculated as:
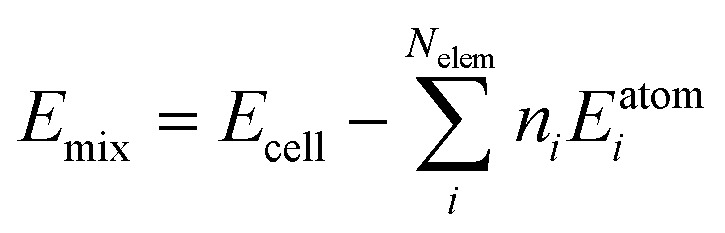
with *E*_cell_ being the total energy of the cell, *E*^atom^_*i*_ being the total energy of a single atom of element *i* in the structure with the lowest calculated energy for the pure elements (fcc for Ir, Pt, Rh and hcp for Os, Ru). *N*_elem_ is the number of elements and *n*_*i*_ is the number of atoms of element *i* in the cell.

### 2.4. ORR measurements in thin film RDE configuration

The ORR activity of the PtIrOsRhRu HEA nanoparticles was determined in oxygen saturated 0.1 M HClO_4_ electrolyte using a thin film RDE configuration.^38,39^ For this a catalyst ink was prepared dispersing the PtIrOsRhRu HEA nanoparticles in a mixture of Milli-Q water and isopropanol (*V*_water_ : V_IPA_ = 3 : 1). To the ink, 1.6 μL mL^−1^ 1 M KOH (aq) was added and then homogenized in a sonicator bath for 10 min. The resulting homogeneous catalyst ink had a total metal concentration of 0.654 g_metal_ L^−1^. Thin catalysts films were prepared by pipetting 9 μL of catalyst ink onto a newly polished glassy carbon (GC) disk, the disk was then dried in an ambient atmosphere under an Ar-gas stream humidified with IPA and H_2_O, leading to the catalyst films with a metal loading of 30 μg_metal_ cm^−2^ for the electrochemical measurements.

### 2.5. *In situ* XRD/XAS studies

Combined XAS and XRD *in situ* experiments were performed at the BM31 beamline (SNBL)^40^ at the ESRF in Grenoble, France. Details on the *in situ* XRD/XAS studies can be found in the ESI,† see Fig. S1. Here, the beamline setup is presented and the data analysis is explained in depth. In short, a 0.7 mm quartz capillary fixed in a U-shaped stainless-steel bracket^41^ functioned as a miniature tube furnace. A gas stream of 5% H_2_ in He was flown through the capillary while heating to 500 °C with a constant heating ramp of 100 K h^−1^. XAS and XRD data were continuously collected during the heating.

## 3. Results and discussion

As discussed in the introduction, the present study aims to investigate the drivers that favor the formation of single-phase HEA nanoparticles at low temperatures. The employed synthesis was first described by Yusenko *et al.*,^42^ where a crystalline, single-source precursor is precipitated from metal salt solutions and thermally decomposed in a reductive gas flow while heating to 500 °C.^29,33,42–44^ The single-source precursor is a complex salt comprised of coordination compounds of anionic and cationic complexes ([M^3+^(NH_3_)_5_Cl][M^4+^Cl_6_]), see also Fig. S2 (ESI†). By introducing variations of M^4+^ = Os, Ir, Pt, and M^3+^ = Ir, Rh, Ru, respectively, the mixing of metals occurs on the local level of the crystalline precursor. Thermolysis in an H_2_-containing gas stream leads to metal reduction, and NH_4_Cl evaporates. Further details on the synthesis and precursor are given in the ESI.† The synthesis approach can yield single-phase HEA nanoparticles^29^ and is thus ideally suited as a model system to study the decisive factors for single-phase formation. We focus on HEAs of five noble metals, Pt, Ir, Rh, Os, and Ru, as they are highly relevant for catalytic applications.^45^ The catalytic significance of these materials is emphasized as the obtained PtIrOsRhRu nanoparticles exhibit improved electrocatalytic performance over Pt for the ORR as demonstrated in Fig. S3 (ESI†). By choosing five noble metals of very similar chemical nature, we attempt to minimalize the mixing enthalpies of the elements. This should avoid a strong preferential formation of either intermetallic phases or phase separation of the individual metals. Apart from PtIrOsRhRu HEA formation, we also analyze the phase formation for a row of bimetallic systems, namely equiatomic mixtures of Ir–Pt, Rh–Pt, Ru–Pt, Ir–Os, Rh–Os, Ru–Os, Rh–Ir, and Ru–Ir (EDX analysis, Fig. S4, ESI†). Keeping the reaction conditions identical throughout all *in situ* experiments, we further eliminate any influences of changing reaction environments. This allows us to investigate the effect of the number of elements in the formation process.

### 3.1. Combined *in situ* structural characterization of bimetallic systems and HEA nanoparticles

**Fig. 1:**
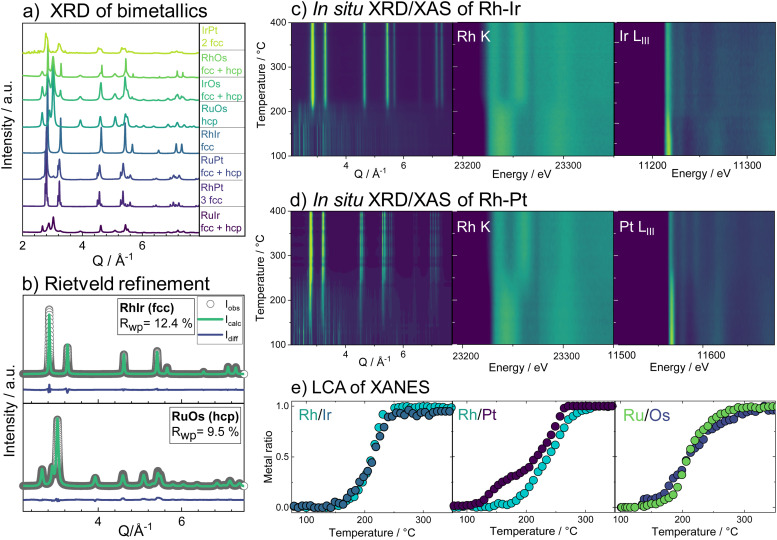
*In situ* studies of alloy formation behavior studied for bimetallic systems. (a) Diffraction pattern of the final product formed (b) Rietveld refinement of the single-phase materials obtained for the Rh–Ir and Ru–Os bimetallic systems. (c) *In situ* formation study of the Rh–Ir fcc alloy showing a contour plot of the diffraction patterns and the Rh K-edge and Ir L_III_-edge X-ray absorption spectra as a function of reaction temperature. (d) Contour plots from X-ray diffraction patterns and Rh-K-edge and Pt L_III_-edge X-ray absorption spectra as a function of reaction temperature, following the phase formation in the Rh–Pt bimetallic system. (e) Metal ratios of the bimetallic samples as determined from LCA fits.

We first consider the products obtained from the bimetallic syntheses, for which XRD patterns are shown in Fig. 1a. The formation of a single-phase alloy is observed only for two out of the eight studied systems: the Rh–Ir system (forming a face-centered cubic (fcc) single-phase), and the Ru–Os system (hexagonal close packed (hcp) single-phase). Rietveld refinements of XRD data from these samples are shown in Fig. 1b confirming the single-phase nature.

In all other cases, at least two separate phases form. Neither the physicochemical properties of the elements (atom radius, ionization energy, electron work function, *etc.*; see ESI,† Table S1) nor the element miscibility^46^ (ESI,† Table S2) can explain the systems that do form single-phase alloys and those that do not. This lack of correlation indicates that single-phase formation may rather be related to the reaction mechanism than to thermodynamics.

A clearer picture arises when studying the phase formation mechanism *in situ* by following the precursor reduction *via* simultaneously recorded XRD and XAS data for all eight bimetallic systems. Fig. 1c and d show the *in situ* data for the Rh–Ir and Rh–Pt systems, respectively. Although Rh–Ir and Rh–Pt are two chemically closely related systems with matching binary phase diagrams,^46^ the samples display very different phase formation behavior. A contour plot of the XRD patterns collected during the synthesis of Rh–Ir is shown in Fig. 1c, allowing us to follow the evolution of crystalline phases during the reaction. The initial crystallization of the fcc phase starts at *circa* 180 °C (see also individual diffraction patterns in the ESI,† Fig. S5) and coincides with the complete reduction of the precursor. During further heat treatment lattice expansion of the Rh–Ir single-phase is observed, as expected for thermal expansion^47,48^ (see lattice parameter from Rietveld refinement in the ESI,† Fig. S6). The simultaneously collected XANES data show changes in the oxidation state of both Ir and Rh (contour plots of Ir L_III_-edge and Rh K-edge, Fig. 1c). It is seen that the reduction of Rh and Ir occurs at the same temperature, simultaneously with the initial alloy crystallization. The Rh and Ir metal precursors reduce in a single step, as shown by linear combination analysis (LCA) of the absorption spectra (Fig. 1e). In contrast, the Rh–Pt sample forms three separate fcc phases. Based on lattice parameters (see ESI,† Fig. S7), the phases correspond to Pt (19 wt%, *a*_3_ = 3.9393(1)) and two Rh–Pt alloys of different compositions; one being very Rh-rich as seen from the refined unit cell parameters (74 wt%, *a*_2_ = 3.8862(1), and 7 wt% *a*_1_ = 3.8271(1) at 500 °C). The contour plot of the temperature-dependent *in situ* XRD patterns (Fig. 1c) reveals the initial crystallization of an fcc phase at 150 °C, while Bragg peaks of the precursor phase are still present up to 250 °C. The lattice parameter of the initially formed phase (*a* = 3.936(1) Å) agrees with Pt. All three fcc phases are present once the reaction temperature reaches 250 °C. The XANES data measured at the Pt L_III_- and Rh K-edges in Fig. 1d show that the reduction of Pt and Rh occurs predominantly at around 250 °C.

The LCA (Fig. 1e) shows a single-step reduction of Rh starting at 190 °C, while Pt starts to be partially reduced already at around 140 °C, concomitant with the crystallization of a primary fcc phase. The initial reduction and crystallization of around one-third of the Pt prevents the formation of a single-phase bimetallic alloy. For the formation mechanism of the Ru–Os single-phase hcp alloy, again a simultaneous, single-step reduction process of Ru and Os is revealed from XANES analysis (Fig. 1e and ESI,† Fig. S8, S9). For all other bimetallic systems (IrPt, IrRu, RuPt, RhOs, IrOs), the reduction process of the two metals occurs either subsequently or involves a two-step reduction (ESI,† Fig. S10–S19). The *in situ* studies of the eight different bimetallic systems show that the formation of a single-phase bimetallic alloy occurs only if the metals in the precursor reduce concomitantly, in a single step at the same temperatures.

With the insights from the bimetallic systems, we now consider the more complex HEA nanoparticles. When all five elements are combined in the single-source precursor, it is possible to form single-phase HEA nanoparticles through the same thermal decomposition process as the bimetals.^29^ By varying the ratio of hcp-phase to fcc-phase metals, the phase formation can be steered to either form single-phase fcc or single-phase hcp HEA particles.^29^ If the metals that crystallize in an fcc structure (Pt, Ir, Rh) are the main component, an fcc alloy is obtained and *vice versa* for the hcp crystallizing metals (Os, Ru). Two HEA materials have been prepared from the set of elements; one where the fcc-forming metals dominate (Ru_0.11_Rh_0.26_Os_0.06_Ir_0.22_Pt_0.35_, fcc-HEA) and one where the hcp-forming metals dominate (Ru_0.31_Rh_0.09_Os_0.30_Ir_0.15_Pt_0.15_, hcp-HEA), composition analysis in the ESI,† Table S2.

**Fig. 2:**
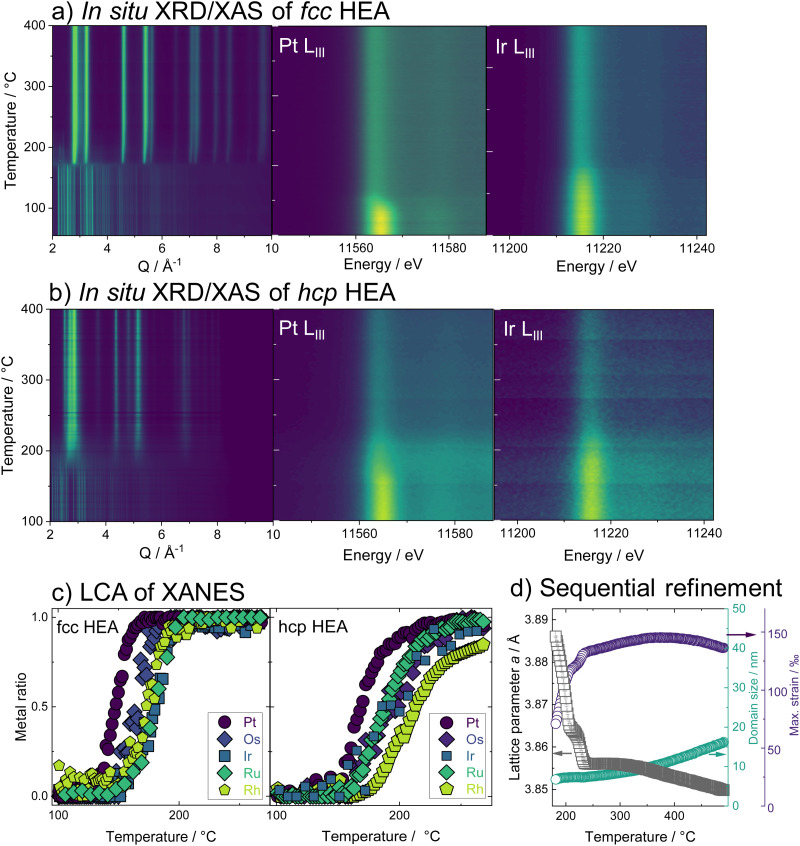
*In situ* studies on the formation of high entropy alloy nanoparticles from five element mixtures. (a) The fcc phase formation and the (b) hcp phase formation with a contour plot of the XRD and the XANES Pt and Ir L_III_-edges during the heating protocol. (c) LCA showing the amount of metal as a function of temperature for the reduction of all individual elements in the HEA samples. (d) Evolution of lattice parameter *a*, crystallite domain size, and lattice strain of fcc-phase HEA as a function of temperature, obtained from sequential Rietveld refinement.

Combining temperature-dependent XRD with XAS data collected *in situ* on absorption edges of all five elements during the reduction, we now track the phase formation in the HEA samples. XRD data of the fcc-phase HEA are shown in Fig. 2a. Crystallization of an fcc phase is observed at 170 °C, while precursor Bragg peaks are still evident until around 230 °C. Moreover, a clear shift in the Bragg peaks to larger diffraction angles indicates a lattice contraction of the fcc phase. The reduction of Pt starts at 150 °C, while Ir reduction occurs at much higher temperatures around *circa* 180 °C, as seen from the L_III_-edge X-ray absorption spectra (contour plots, Fig. 2a). The LCA of the XANES of all five elements, as shown in Fig. 2c, highlights this fact: the metals reduce stepwise in the five-element mixture. Pt reduces at the lowest temperatures, followed by Os, Ir, Ru, and lastly Rh. The lattice constant obtained from sequential Rietveld refinement of the *in situ* XRD data is in good agreement with the reduction order seen by XANES. The refined lattice parameter of the initially formed phase is slightly smaller than pure Pt, likely due to Ir and Os inclusion. The fcc lattice contracts upon further reduction of all elements (Fig. 2d, black curve) and subsequent incorporation of metals with smaller atomic radii into the alloy. Heating the sample after complete precursor reduction leads to further lattice contraction. The initial lattice contraction of the fcc phase at temperatures below 250 °C is accompanied by a pronounced increase in lattice strain (Fig. 2d, purple). At the high-temperature contraction above 350 °C, the strain remains almost constant, and the crystallite domains grow (Fig. 2d, green curve, see also ESI,† Fig. S20–S22.) The *in situ* data that follow the formation of the hcp single-phase HEA, shown in Fig. 2b, displays a similar picture. The HEA particles form by stepwise reduction of the metals as well, again starting with an initial reduction of Pt. Crystallization of a metallic phase that appears defect-rich and of very small domain size is evident at around 200 °C, where a large fraction of the elements is already fully or majorly reduced. During the following heating, the crystal lattice of the hcp phase expands (see ESI,† Fig. S23–S25).

### 3.2. *Ex situ* structural characterization of fcc & hcp HEAs

Having studied the HEA formation mechanism based on combined *in situ* XRD and XAS, we now characterize the synthesized HEAs in detail. The atomic structure at room temperature of HEA nanoparticles, prepared by the low-temperature reduction with H_2_ in Ar in a conventional tube furnace, is analyzed. We find the same phase formation behavior of the bimetals and HEA sample in the *ex situ* as in the *in situ* experiments, despite differences in reaction conditions between the two sets of experiments. With Rietveld refinement, a lattice parameter *a* = 3.8324(1) Å is determined for the fcc-phase HEA, which is in good agreement with the expected lattice constant for a single-phase fcc alloy of the composition determined by EDX analysis (ESI,† Table S3). For the hcp alloy, lattice constants *a* = 2.7183(2) Å and *c* = 4.3317(4) Å are obtained from the refinement. The refinements cannot correctly describe the ratios of Bragg peaks, which indicates a very distorted^49^ and defect-rich structure. The refinement of both the XRD and PDF data of the hcp sample was improved when adding an fcc phase of very small particle size to the model. As we find a large number of planar defects in the HR-TEM analysis as well as an even distribution of elements in the sample, we attribute the fcc contribution rather to the presence of stacking faults in the material than a separate crystallographic phase (see ESI,† Fig. S26–S29). X-ray total scattering data combined with pair distribution function (PDF) analysis of both single-phase HEA samples shows pair distances up to only around 30 Å (fcc) and 50 Å (hcp). This fast damping is expected for disordered lattices^50^ and confirms the strained nature of the crystal lattice of the HEA nanoparticles.

**Fig. 3:**
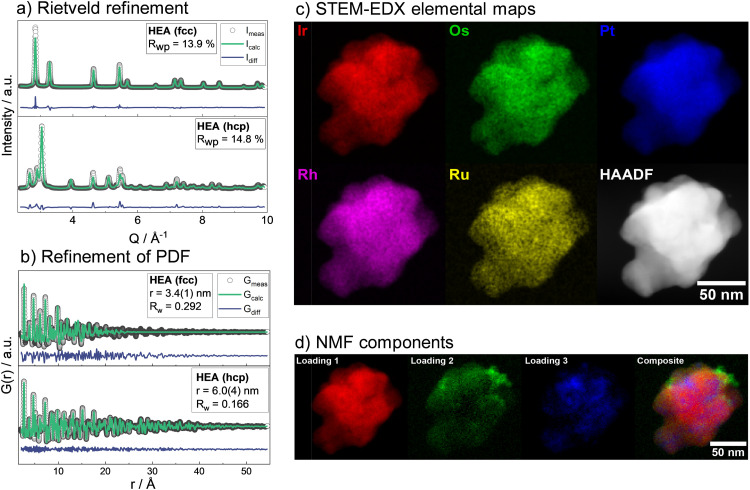
*Ex situ* characterization of high entropy alloy nanoparticles with (a) Rietveld refinement of XRD patterns and (b) PDF from X-ray total scattering data with refinement. Data of the hcp-HEA sample was refined with a two-phase model including a contribution from an fcc-phase (c) the element distribution in the particles of the fcc-HEA is visualized for the individual metals by scanning transmission electron microscopy with energy-dispersive X-ray spectroscopy (STEM-EDX) with high-angle annular dark-field (HAADF) micrograph (d) individual loadings picked up by non-negative matrix factorization (NMF).

The atomic structure of the individual HEA particles is also investigated by scanning transmission electron microscopy combined with energy-dispersive X-ray spectroscopy (STEM-EDX). Based on the elemental distribution maps created from STEM-EDX measurements for the fcc-phase HEA, shown in Fig. 3c, the spatial distribution of all elements within this one particulate appears completely and equally mixed. However, upon more detailed analysis, *i.e.*, by using non-negative matrix factorization (NMF), there appears to be some segregation of different elements in various areas. The area highlighted in red in Fig. 3d shows the most dominant NMF loading, which contains all five elements. This indicates that a major fraction of the particle shows complete mixing between the five elements. The green loading, however, highlights areas containing all elements except for Pt. Finally, the blue loading shows areas rich in Pt, containing some Rh, less Ru, and Os, while little to no Ir is found here (ESI,† Fig. S30). As seen in the composite image, it is evident that this separation occurs on the nanoscale. There is thus still a significant amount of mixing present and the demixing only becomes visible upon advanced analysis. While STEM-EDX analysis confirms an overall mixing of all elements in the HEA particles, the NMF analysis shows minute fractions of local segregation in specific areas on the nanoscale. This is predominantly related to an uneven distribution of Pt on a local level, with areas that are either higher (blue) or lower (green) in Pt. We can relate this finding to the particle formation mechanism.

As platinum is reduced at the lowest temperature and dominates the initial crystallization, such an uneven Pt distribution can be linked to remnants of Pt-rich nuclei.

### 3.3. Simulated particle formation and influence of number of elements

The combined data analyses from XRD and XAS evidence clearly that the elements in the five-metal precursor reduce stepwise. However, a single-phase alloy forms nonetheless. This behavior contrasts with the bimetallic case, suggesting that the rules governing phase formation differ for the systems containing two or five elements: Single-phase alloy formation occurs more readily if more elements are present, as systems that are immiscible on the bimetallic level can be driven to mix by introducing more elements. As the resulting phases of the binary particles cannot readily be explained by their thermodynamic stability, we devised a kinetic model to rationalize the experimental results based on the restrictions imposed by the synthesis. When comparing five-element and two-element mixtures, the concentration of individual atom types is reduced the more elements are present. This will make it increasingly difficult for elements with strong preferences of ordering to form an intermetallic structure or for elements that repulse each other to segregate, simply because they are more dilute. The atoms are further limited in their free mobility in the solid precursor, and the mixing of elements in the precursor leads to spatially separated nucleation centers of the initially reduced elements, *e.g.*, platinum.

**Fig. 4:**
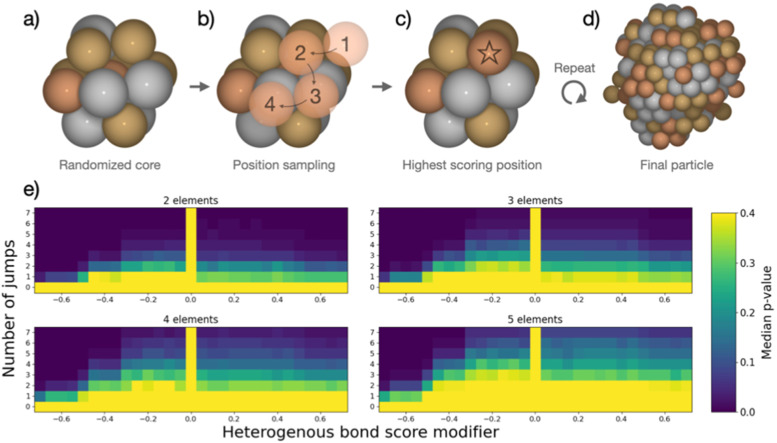
(a) Initial 13-atom core composed of randomized elements. (b) Random element from the pool of available atoms is added in a random position and allowed to sample potential atomic positions for a variable number of jumps. (c) The atomic position with best score is chosen as final position. (d) This process is repeated until the pool of available atoms is empty and the final particle size of 250 atoms is reached. (e) For each set of parameters, 500 particles were simulated and analyzed as described in the text, yielding a median *χ*^2^-value as described in text. Compared to the *χ*^2^-distribution of random particles a *p*-value for each set of parameters was calculated and are displayed here as heat maps.

To quantify the increased likelihood of forming random particles in multi-metallic samples, we simulate particle formation and perform a statistical analysis. A model was devised to visualize the effect that the number of elements in the simulation has on particle formation. Based on the attributes imposed by the synthesis, the devised model does not seek to simulate particle formation accurately but rather mimics the principles of restricted atom migration from a random onset. Using this model coupled with statistical analysis, we can quantify the increased likelihood of forming random particles by increasing the number of elements. We simulate nanoparticle formation by starting from a 13-atom core in an fcc structure (Fig. 4a), continuously adding single atoms to randomly picked surface sites of the particle. Both the initial core atoms and the individually added atoms were randomized, and all atoms were drawn from a pool of available atoms to achieve equimolar particle composition. Two adjustable parameters, apart from the number of elements, were included in the simulation. (I) The added atom was allowed to jump to adjacent atomic positions on the particle surface for an adjustable number of iterations (Fig. 4b). This mimics the kinetic energy of the atom to find the most stable configuration before losing its kinetic energy. We evaluate the stability of different atomic positions by giving each position a score based on how many bonds the added atom could make to the particle. (II) The score of each position was adjusted based on how many heterogeneous bonds were formed. This represents the mixing enthalpy between atoms. Element mixtures with favorable mixing enthalpy were simulated by increasing the scores of heterogeneous bonds, while segregation-prone mixtures were simulated by reducing the bond score. The simulations included the identity of the elements but no unique inter-element interactions, reducing the interactions to either homogeneous or heterogeneous bonds. Based on these rules, the new atom was added in the position with the highest score (Fig. 4c). Atoms were continuously added following this procedure until reaching a particle size of 250 atoms (Fig. 4d). Pearson's *χ*^2^ test statistic was used to quantify the stochastic composition of a particle by the number of times each unique bond occurred as compared to its expected number of occurrences if the two atoms forming every bond were chosen randomly with equal probability. For each set of adjustable parameters, 500 simulations were performed, which yielded a median *χ*^2^-value. From this value, a *p*-value was calculated as the fraction of 10 000 randomly configured particles with the same number of elements, that had a higher *χ*^2^-value than the median value. The resulting *p*-value was used to construct the heat maps in Fig. 4e. A detailed description is given in the ESI,† Fig. S31–S34.

In the heat maps, the two adjustable parameters, (I) number of jumps and (II) modification of the bond score for heterogeneous bonds, are shown on the *y*- and *x*-axis, respectively. Parameter sets with a low median *p*-value (marked blue) correspond to a low likelihood of the particles being randomly configured, as the observed ratios of bonds are skewed from the expected ratios. Contrarily, the bright areas with high *p*-value correspond to a situation where we cannot exclude the particles from being random as the observed bond ratios are close to the expected. The heat maps for particle formation include two extreme cases where random particle formation can occur: (1) the added atom is not allowed to move on the surface (zero jumps). The particle is by definition random as randomly picked atoms are added at a random surface position. (2) The mixing enthalpy is zero (zero bond score), so there is no interaction between different elements. Without any preferential binding, the atoms are added at a random position, also resulting in a random structure. Random particle formation is excluded by two other extreme cases: (1) strong attraction between heteroatoms (high heterogeneous bond score) leads to the formation of an intermetallic phase, resulting in fewer homogenous bonds than would be observed in a random particle. (2) Strong repulsion between different elements (low heterogeneous bond score) leads to clustering of one atom type, *i.e.* segregation. Between these four extreme cases, the simulated particles with different sets of parameters exhibit a continuum of observed median *p*-values forming the gradients in heat maps (Fig. 4e). We observe that, as more elements are included in the simulation, more and more particles cannot be excluded from being random as the *p*-values increase for the majority of parameter sets.

This simple model of particle formation demonstrates that adding more elements innately promotes a more random nanoparticle, regardless of element interactions. Naturally, this model offers an idealized perspective and does not reflect all experimental details. In reality, there will be distinct mixing enthalpies between the elements. This will introduce a preference for certain structures which is opposed by the limited mobility of atoms. This explains how a rather mixed particle is obtained in the synthesis for a five-element mixture, which is restricted in the binary case, but also sees signs of local segregation in the NMF analysis. The model relates well to the single-source precursor synthesis, where a solid precursor is reduced at moderate temperatures, hence limiting the free mobility. Extending this approach to other syntheses with similar solid, mixed precursors can thus provide a viable way to design new low-temperature approaches for small high entropy alloy nanoparticles. It can further be argued that starting with a high number of different elements favors the exploration of high-entropy material spaces.^51^ In solution-based synthesis where the mobility of species in solution is much less restricted than in the solid state, the formation of single-phase nanoparticles is expected to be more difficult and other factors may dominate the particle phase formation.

## 4. Conclusions

Simultaneous *in situ* X-ray diffraction and multi-edge X-ray absorption spectroscopy experiments allowed us to study the formation mechanism of noble metal bimetallic and high entropy alloy nanoparticles in depth. The samples were prepared by reduction of single-source precursors. For five-element mixtures, the formation of a single phase occurs more readily than in the binary samples. Under the studied reaction conditions, single-phase formation for binary alloys was restricted to systems where cooperative reduction occurred, while single-phase HEA particles could be obtained even though the elements were reduced consecutively. The findings are explained with a stochastic model that simulates the particle formation process. Regardless of the element interactions, the model quantifies that it is more likely to form a random alloy if more atom types are present in a system with limited free atom mobility. This highlights that knowledge of the formation mechanism is crucial for understanding the material formation pathways. Extending this to other syntheses with similar solid, mixed precursors can provide a way to design new low-temperature approaches for small high entropy alloy nanoparticles. A high number of different elements, therefore, promotes the exploration of high-entropy material spaces for catalytic applications. Structural characterization of the particles reveals that the lattices are very strained and the HEA nanoparticles contain a high number of defects. Detailed STEM-EDX analysis combined with component analysis of the images uncovers a limited separation of specific element types on a local level, which is related to the step-wise reduction and crystallization process. This demonstrates that while bulk techniques indicate the formation of a random high entropy alloy, advanced microscope analysis is needed to confirm or refute the particles' true randomness.

## Conflicts of interest

There are no conflicts of interest to declare.

## Supplementary Material

EY-001-D3EY00201B-s001
