# Supplementary material for: The more the better: on the formation of single-phase high entropy alloy nanoparticles as catalysts for the oxygen reduction reaction
Source: EES Catal. 2023 Aug 22;1(6):950–60. doi: 10.1039/d3ey00201b (PMC10621632; doi:10.1039/d3ey00201b)
Supplement: EY-001-D3EY00201B-s001 [file EY-001-D3EY00201B-s001.pdf]

Electronic Supplementary Material (ESI) for EES Catalysis.
This journal is © The Royal Society of Chemistry 2023

Supplementary Materials for

The more the better: on the formation of single-phase high entropy alloy nanoparticles as catalysts for the oxygen reduction reaction

Rebecca K. Pittkowski,[1,*] Christian M. Clausen,[1] Qinyi Chen,[1] Dragos Stoian,[2] Wouter van Beek,[2] Jan Bucher,[3] Rahel L. Welten,[3] Nicolas Schlegel,[3] Jette K. Mathiesen,[1,4] Tobias M. Nielsen,[1] Jia Du[3], Asger W. Rosenkranz,[5] Espen D. Bøjesen,[5] Jan Rossmeisl,[1] Kirsten M. Ø. Jensen,[1,*] and Matthias Arenz[3,*].

[1] Center for High entropy alloy catalysis (CHEAC), Department of Chemistry, University of Copenhagen, Copenhagen, Denmark
[2] Swiss Norwegian Beamline, European Synchrotron Radiation Facility (ESRF), Grenoble, France
[3] Department of Chemistry, Biochemistry and Pharmaceutical Sciences, University of Bern, Bern, Switzerland
[4] Department of Physics, Technical University of Denmark, Kgs. Lyngby, Denmark
[5] Aarhus University, Interdisciplinary Nanoscience Center, Aarhus, Denmark

Corresponding authors: Rebecca.pittkowski@chem.ku.dk, kirsten@chem.ku.dk, matthias.arenz@unibe.ch

The PDF file includes:

Materials and Methods
Supplementary Text
Figs. S1 to S32
Tables S1 to S16
References

Materials and methods

In situ XRD/XAS experiment - beamline set-up

Combined operando X-ray absorption (XAS) and XRD experiments were performed at the BM31 beamline (SNBL)[1] at the ESRF in Grenoble, France. The single-source precursor in powder form was loaded into a quartz capillary of 0.7 mm in diameter from Hilgenberg GmbH (Germany). Quartz wool plugs were used to secure that the precursor powder remains in place, while gas is flown through the capillary. The capillary was fixed into a U-shaped stainless-steel bracket with swage-lock connections for gas flow and glued at the two ends to ensure a leak-proof system (see **Figure S1**).[2] The gas flow (5% H_2 in He) through the capillary was aided by a series of Bronkhorst mass flow controllers (MFCs) calibrated before the start of the experiment. The samples were heated to the desired temperature of 500 °C with a ramp of 100 K/ h by using a heat gun run by a Eurotherm 2400 controller. XAS-XRD data were collected in a quasi-simultaneous manner using a Si(111) double crystal monochromator (DCM). XAS spectra were acquired at the Os (L_{III}, 10.8709 keV), Ir (L_{III}, 11.2152 keV), Pt (L_{III}, 11.5637 keV), Ru (K, 22.1172 keV), and Rh (K, 23.2199 keV) edges in fluorescence mode using a Vortex one-element silicon drift detector (SDD) and FalconX electronics for extreme high count rate performance. Corresponding metal foils were used for the energy calibration (measurement performed in transmission geometry). XRD data were collected with a 2D DEXELA CMOS (complementary metal-oxide semiconductor) pixel detector at an energy of 36.617 keV setting the wavelength to 0.33895 Å. Wavelength calibration was achieved by measuring a standard lanthanum hexaboride (LaB6, NIST SRM 660b) powder samples in a 0.2 mm glass capillary.

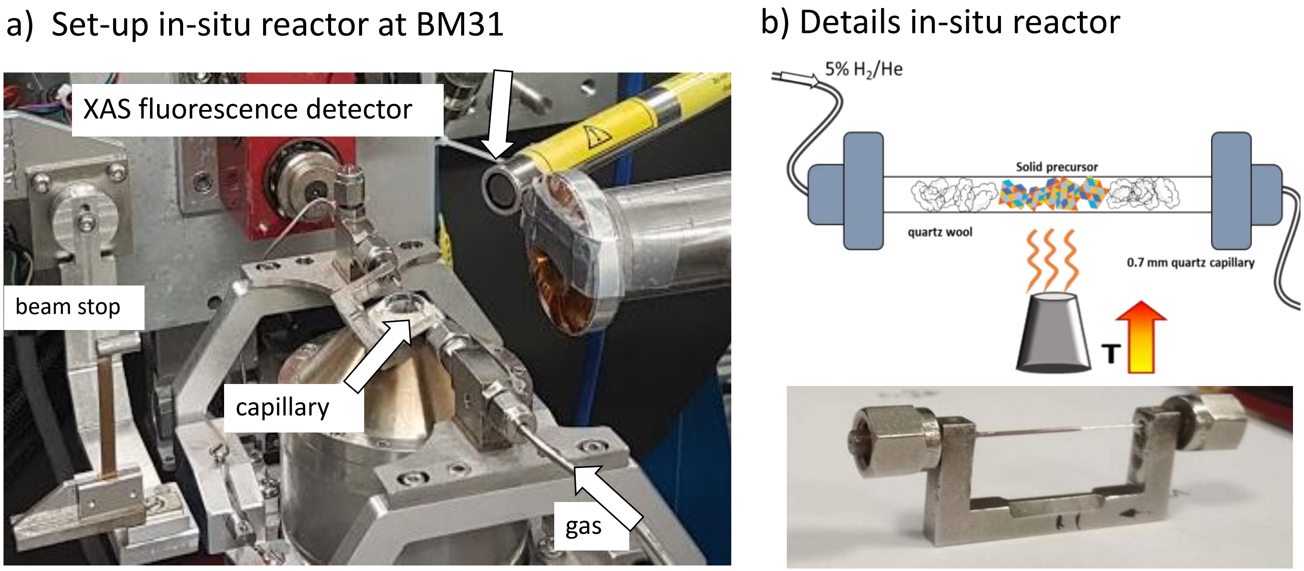

Fig. S1 a) Photograph showing the general beamline set-up with the *in situ* reactor put in place detailing the components of the flow-through reactor. The 2D powder diffraction detector is placed left of the beam stop (not in photograph) b) schematic drawing of the flow-through *in situ* reactor and photograph of an assembled reactor.

Data analysis

The detector distance and geometrical parameters for XRD analysis were obtained from calibration in Fit2D[3] using a LaB_6 standard sample. The 2D scattering images collected for the *ex situ* samples were azimuthally integrated using pyFAI[4] in Dioptas[5]. *In situ* diffraction datasets were integrated at the beamline with the Bubble client[6] based on pyFAI.

Rietveld refinements of powder diffraction patterns of both *ex situ* and *in situ* diffraction data were performed using the FullProf[7] suite program package. The peak profiles were described by Thompson-Cox-Hastings pseudo-Voigt functions[8]. Instrumental peak broadening was accounted for through the analysis of a NIST standard LaB_6 sample. For the high entropy alloy materials (five-metal mixture) the apparent size and max. microstrain contribution to peak broadening was extracted from refinable parameters of the FWHM (U, I_G, X, and Y) using FullProf, as reported by Rodríquez-Carvajal & Roisnel[9]. The parameters I_G and Y describe the Gaussian and Lorentzian contributions of isotropic size broadening, while the parameters U and X describe the Gaussian and Lorentzian contributions of isotropic microstrain broadening, respectively. Due to the different angular dependence of microstructural parameters on the FWHM of diffraction peaks, it is possible to separate the size and strain contributions[10]. The apparent sizes (Å) and max strain values (‰) are reported as averages, determined from the values calculated for each reflection. The data were fit with models of the metallic close-packed lattices; *fcc* (Fm-3m, 225) or *hcp* ($P6_3$/mmc, 194).

For the X-ray total scattering data, the Kapton capillary background was subtracted from the data before obtaining the PDF. The total scattering data were Fourier transformed to obtain the PDF with xPDFsuite using PDFgetX3[11] and modeled using PDFgui[12]. The PDF refinements were done in the r-range 1.6-60 Å. Lattice parameters and crystallite sizes of the samples were obtained from real-space Rietveld refinement by taking into account instrumental PDF damping (Q_{damp} parameter), which was determined from a LaB_6 standard using total scattering data measured in the same instrumental configuration.

Data treatment and analysis of the X-ray absorption spectra was performed with the ATHENA[13] and LARCH[14] program packages. The raw spectra were energy aligned to the respective metal reference foil, background corrected, and normalized by the edge step. The XANES data were analyzed with linear combination analysis (LCA). Precursor spectra, as well as spectra of the formed alloys, were pre-selected as the respective components and the LCA was performed on the normalized XANES data with the restriction that the weights of the components add to one.

Precursor structure

The XRD patterns obtained from the single-source precursor powders were 'spotty', indicating the presence of large single crystallites, which complicates the structural analysis of the precursor structures. A Le Bail fit of the *fcc*-HEA single-source precursor structure is shown below, using the monoclinic crystal structure (C 2/m) of the binary complex salt $[Ir(NH_3)_5Cl]_2[OsCl_6]Cl_2$ as a reference[15] (ICSD: 98112). The fit is not able to fully describe the relative intensities of the

individual Bragg peaks, which can be explained by the grainy nature of individual diffraction rings in the precursor powder, see 2D detector image on the right, which leads to additional intensity of individual Bragg peaks in the integrated diffraction pattern. It indicates, nonetheless, that the precursor can most likely be described as a single phase, which agrees with that reported in the literature[16]. The obtained lattice parameters deviate, however, from those of the two-metal parent structure, indicating that the five-metal mixed precursor salt has a different lattice than the reference.

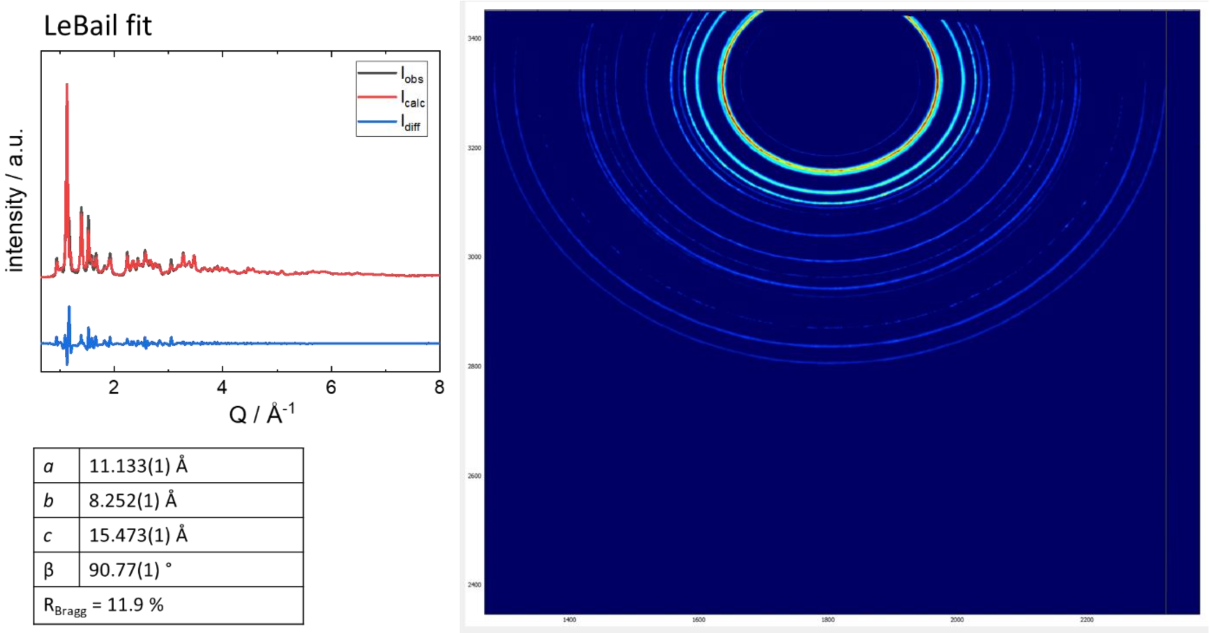

Fig. S2 Le Bail fit of the single-source precursor powder of the *fcc*-HEA sample used in the *in situ* study, fit with a model based on the monoclinic literature precursor $[Ir(NH_3)_5Cl]_2[OsCl_6]Cl_2$ (ICSD: 98112).

<u>Thermodynamic parameters of metals</u>

Table S1. Important physicochemical properties of the noble metals. The electron work function value is given for polycrystalline materials unless otherwise indicated, and was determined by the photoelectric effect. The sources for the tabulated values are given in the respective row.

	**Ru**	**Rh**	**Os**	**Ir**	**Pt**
**Atom radius [Å]**[17]	1.34	1.35	1.35	1.36	1.39
**Crystal structure**[17]	*hcp*	*fcc*	*hcp*	*fcc*	*fcc*
**Lattice parameter**[17]					
***a* [Å]**	2.71	3.80	2.74	3.84	3.92
***c* [Å]**	4.28	N/A	4.32	N/A	N/A
**Cohesive energy [eV/ atom]** [17]	6.74	5.75	8.17	6.94	5.84
**Ionization energy [eV]** [17]	7.36	7.46	8.70	9.00	8.96

**Surface energy [mJ/m^2]**[18]	3050	2700	3450	3000	2480
**Electronegativity χ [Pauling scale]**[19]	2.2	2.3	2.2	2.2	2.2
**Melting temperature [K]**[19]	2333	1963	3033	2446	1768
**molar heat capacity [J/mol*K]** [19]	24.1	25.0	24.7	25.1	25.9
**Electron Work function Φ [eV]** [19]	4.71	4.98	5.93	5.67 (100)	5.64

Table S2. Mixing energy determined from DFT calculations for all investigated systems in kJ/mol with standard deviation reported in parenthesis.

**Alloy**	E_{mix}^{fcc}	E_{mix}^{hcp}	**Phases formed**
**IrOs**	2.85(1.60)	1.76(1.26)	*fcc* + *hcp*
**IrPt**	5.61(0.74)	13.67(1.29)	2 *fcc*
**IrRh**	-1.37(0.13)	3.75(0.50)	*fcc*
**IrRu**	-1.99(1.47)	-3.39(1.79)	*fcc* + *hcp*
**PtRh**	-0.79(0.22)	5.10(0.22)	3 *fcc*
**PtRu**	2.13(1.26)	7.51(1.36)	*fcc* + *hcp*
**OsRh**	7.84(1.47)	5.78(1.30)	*fcc* + *hcp*
**OsRu**	11.38(0.21)	-0.47(0.11)	*hcp*
$\mathbf{Ru_{0.11}Rh_{0.26}Os_{0.06}Ir_{0.22}Pt_{0.35}}$	1.79(1.24)	-	*fcc*
$\mathbf{Ru_{0.31}Rh_{0.09}Os_{0.30}Ir_{0.15}Pt_{0.15}}$	-	8.83(2.62)	*hcp*

ORR measurements of PtIrOsRhRu HEA nanoparticles in thin-film RDE configuration

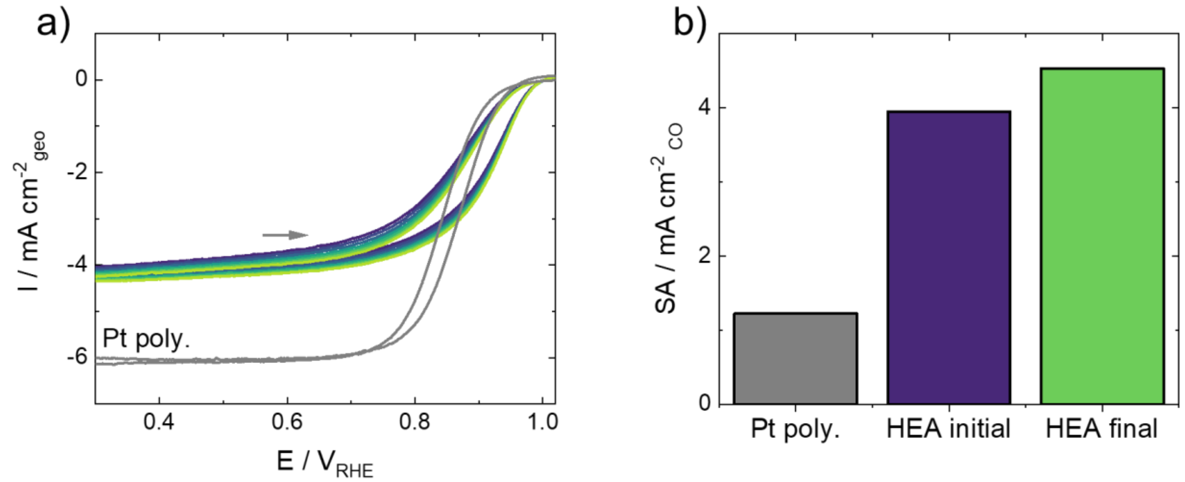

Fig. S3 a) Cyclic voltammograms (CVs) of *fcc*-HEA sample (25 cycles, going from dark to bright) compared to a polycrystalline Pt electrode recorded in oxygen saturated 0.1 M $HClO_4$ electrolyte with a scan rate of 50 mV s^{-1} and a rotation speed of 1600 rpm and b) comparison of the electrochemical ORR performance of the HEA NPs evaluated for the 2nd CV (HEA initial) and the final CV (HEA final) compared to the polycrystalline Pt reference. The specific activity (SA)[20] determined at 0.9 V vs RHE from the positive-going potential sweep is normalized to the electrochemically active surface area (ECSA) of the catalysts determined from CO stripping experiments.

Studies of bimetallic nanoparticles

<u>EDX analysis of bimetallic samples</u>

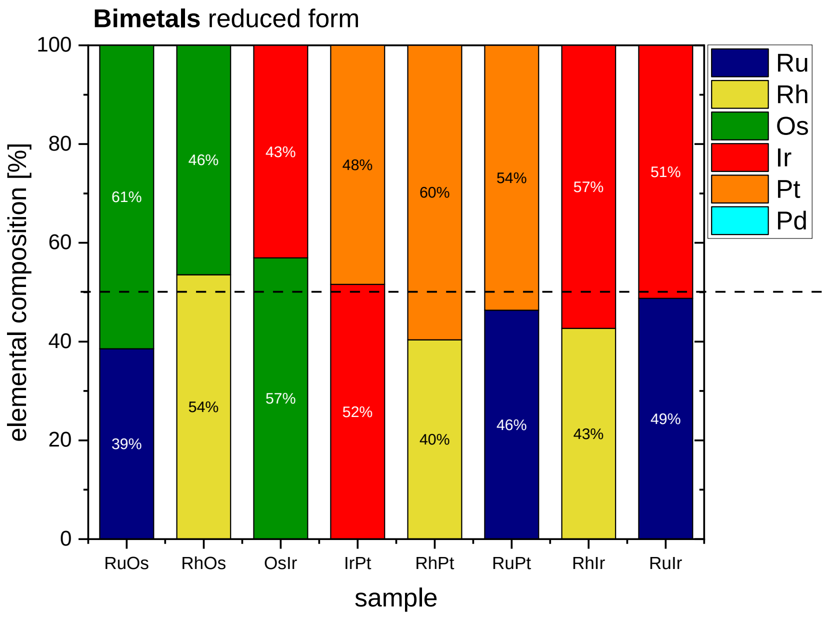

Figure S4 Elemental composition of the bimetallic nanoparticle powder samples as determined by Energy-dispersive X-ray spectroscopy (EDX) analysis.

The discrepancies found between the nominal compositions (50:50 for all bimetallics) and the EDX-determined compositions can be explained by the synthesis method used. The synthesis includes the precipitation of the single-source precursor from the hot solution containing both the anionic and cationic metal precursors. Small compositional deviations occur in the precipitated single-source precursor.[16] Different amounts of residual metals remain in the supernatant, and we find a surplus of Pt (22 ppb) compare to Ir (17 ppb) by ICP-MS analysis of the supernatant.

Individual frames from in situ XRD data collected of bimetallic systems

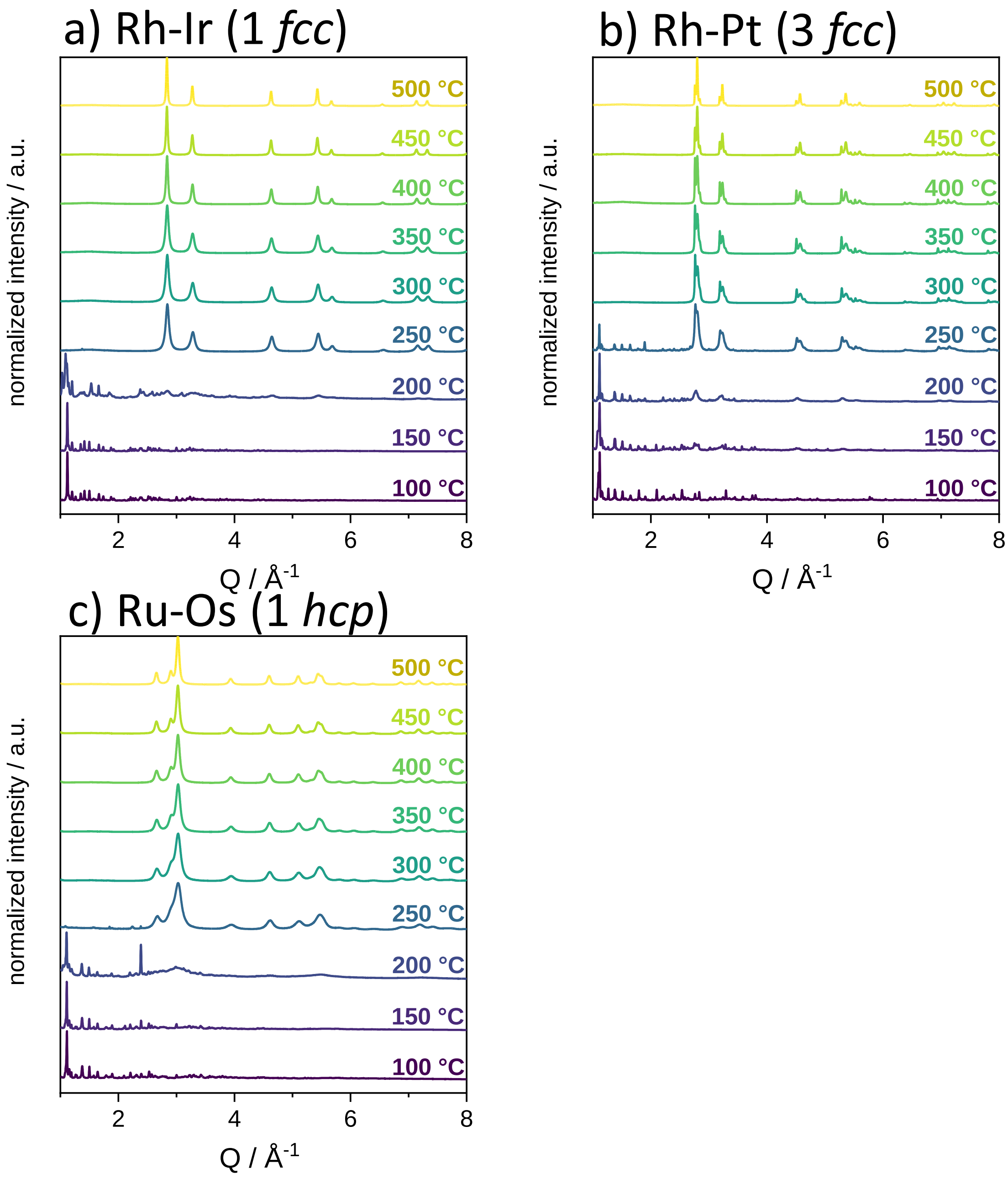

Figure S5 XRD patterns taken at different temperatures during the reductive heating showing the gradual precursor reduction and crystallization of metallic particles a) in the Rh-Ir bimetallic system for formation of a single *fcc* phase, b) in the Rh-Pt bimetallic system for the formation of separate *fcc* phases (three phase system), and c) in the Ru-Os bimetallic system for the formation of a single *hcp* crystalline phase.

Rh-Ir bimetallic system

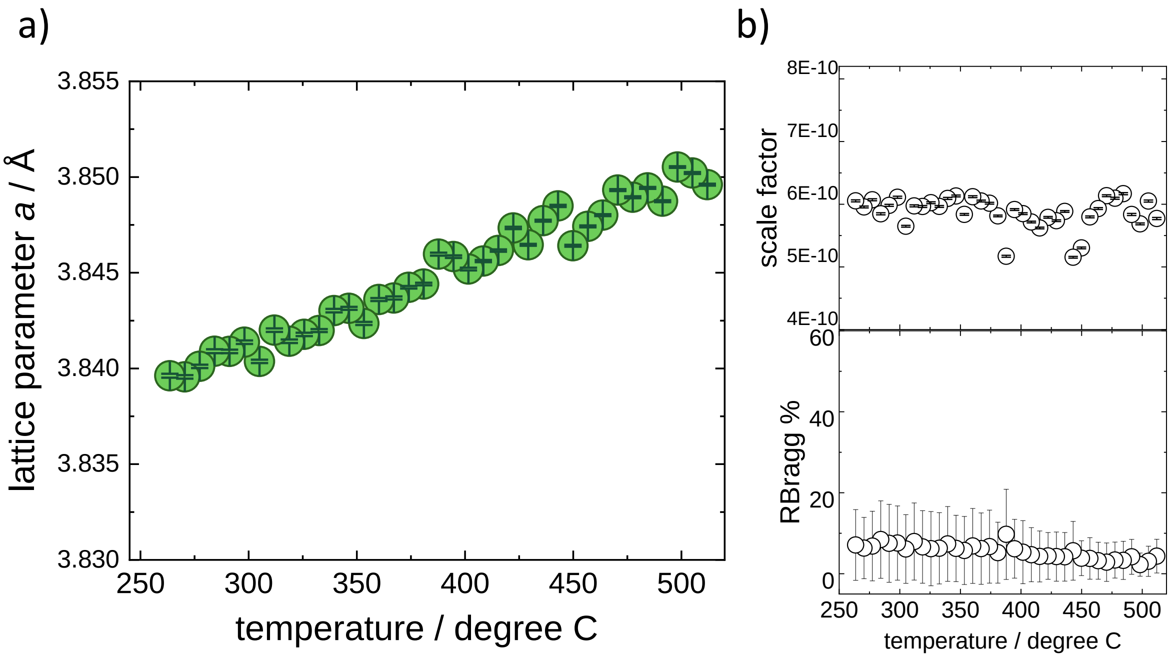

Figure S6 Results obtained for the sequential Rietveld refinement of the in-situ diffraction data of the IrRh alloy showing a) the evolution of lattice parameter a of the *fcc* phase (225, Fm-3m) as well as b) the scale factor and R_{Bragg} values as a function of temperature in the *in situ* experiment.

Rh-Pt bimetallic system

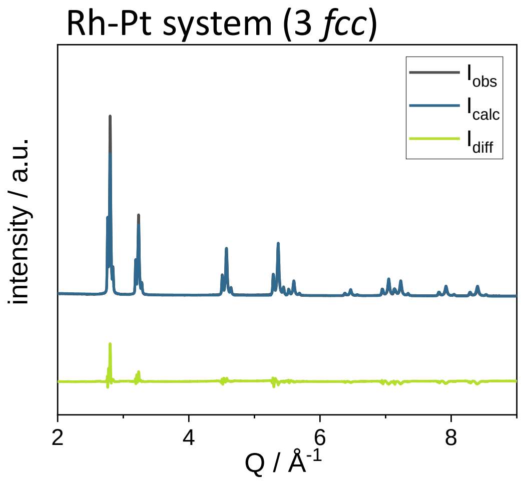

		R_{wp} = 19.8 %
fcc - 1	*a* = 3.8862(1) Å	
	V = 58.69(1) Å^3	74.1(4) wt.%
fcc - 2	*a* = 3.9393(1) Å	
	V = 61.13(1) Å^3	19.0(2) wt.%
fcc - 3	*a* = 3.8271(1) Å	
	V = 56.05(1) Å^3	6.9(2) wt.%

Figure S7 Rietveld refinement results with lattice parameters obtained for the final frame at 500 °C of the *in situ* study within the Rh-Pt bimetallic system, for a three-phase mixture with three *fcc* phases (225, Fm-3m).

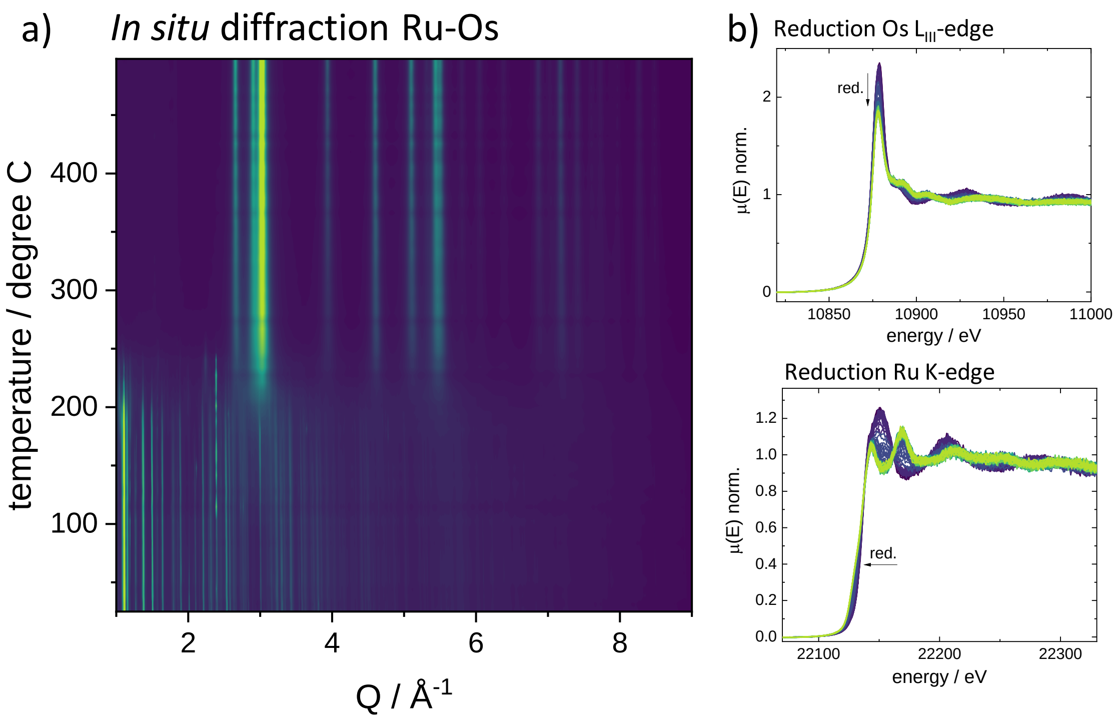

Figure S8 *In situ* XRD/XAS study of phase formation in the Ru-Os bimetallic system. a) Contour plot of the *in situ* X-ray diffraction data collected during reductive heating. b) X-ray absorption spectra (XANES) of the Os L_{III}-edge and Ru K-edge during the reductive heating to follow the reduction of the individual elements. Arrows indicate the changes during reductive heating.

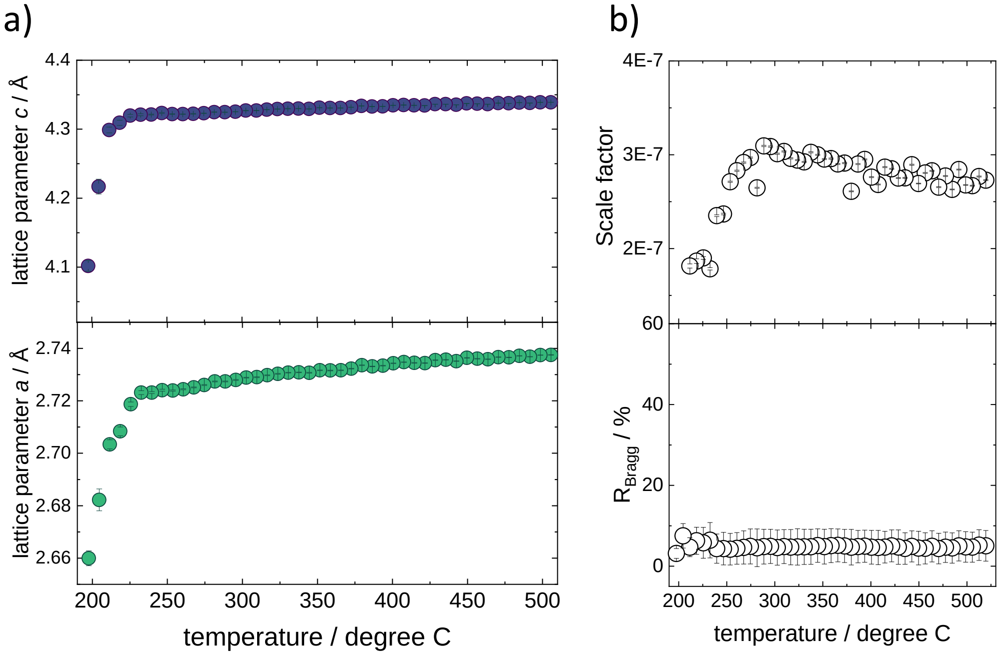

Figure S9 Results obtained for the sequential Rietveld refinement of the *in situ* diffraction data showing a) the lattice parameters *a* and *c* of the *hcp* phase (194, P63/mmc) as well as b) the scale factor and R_{Bragg} values as a function of temperature in the *in situ* reactor.

Os-Ir bimetallic system

For the Os-Ir bimetallic system, a mixture of an *hcp* and *fcc* phase is obtained (see **Fig. S9**), largely agreeing with the formation of pure osmium *hcp* and iridium *fcc* particles. The fractions of the *fcc* phase and *hcp* phase as determined by Rietveld refinement are in good agreement with the composition as determined by EDX (43 at.% Ir and 57 at.% Os). Similarly, the lattice parameters of the obtained *fcc* phase and *hcp* phase agree well with the literature: Ir (a=3.8445 at 500 °C)[21] and Os (a=2.7368 °C, c=4.3225 Å at 500 °C)[22]. The refinement is not able to describe correctly the intensities of certain Bragg peaks, which can be ascribed to a highly defective nature of the particles formed (see discussion in the main text).

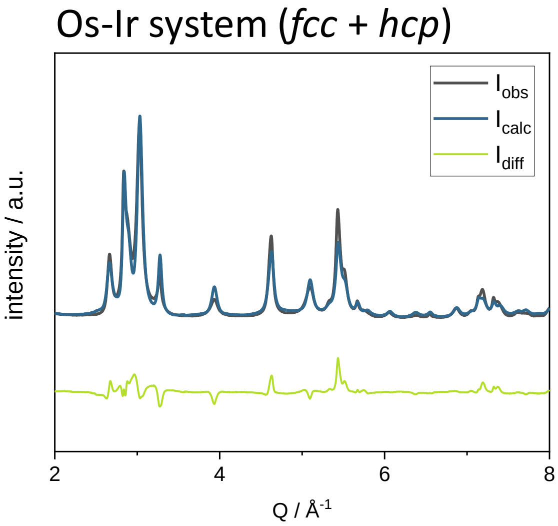

		R_{wp} = 21.1 %
fcc	*a* = 3.8351(7) Å	
	V = 56.41(2) Å^3	44.0(6) wt.%
hcp	*a* = 2.7233(4) Å	*c* = 4.3380(8) Å
	V = 27.86(1) Å^3	56.0(7) wt.%

Figure S10 Rietveld refinement with lattice parameters obtained for the final frame at 500 °C of the *in situ* study within the Os-Ir bimetallic system, for a two phasic system with one *fcc* phase (225, Fm-3m) and one *hcp* phase (194, $P6_3/mmc$).

The *in situ* mechanistic study (**Fig. S11**) shows that a step-wise phase formation occurs when the *hcp* phase starts to crystallize at around 220 °C followed by the crystallization of the *fcc* phase at slightly higher temperatures (onset around 260 °C). Analysis of the XANES data shows that the reduction of Os and Ir occurs stepwise. Iridium is reduced in a single step starting at around 230 °C. For osmium, however, an intermediate is found (formation onset 100 °C) which is followed by the reduction to the metallic state that starts at circa 180 °C. The reduction of Os at lower temperatures than the reduction of Ir agrees with an initial crystallization of the *hcp* phase that seems to be predominantly osmium. This is followed by the crystallization of the Ir-dominated *fcc* phase, with iridium reducing at higher temperatures than Os.

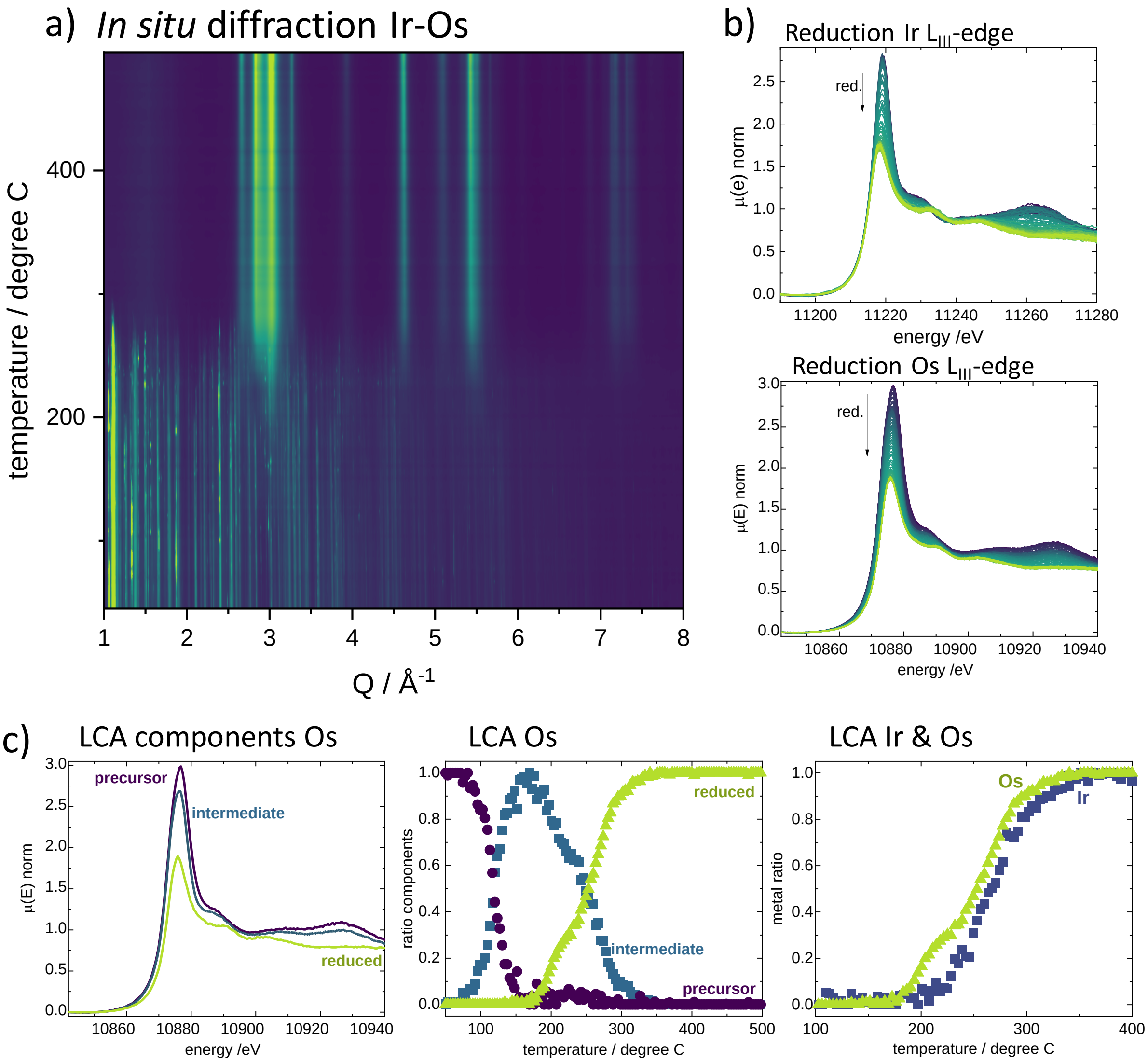

Figure S11 *In situ* XRD/XAS study of phase formation in the Os-Ir bimetallic system. a) Contour plot of the i*n situ* X-ray diffraction data collected during reductive heating. b) X-ray absorption spectra (XANES) of the Ir L_{III}-edge and Os L_{III}-edge during the reductive heating to follow the reduction of the individual elements. Arrows indicate the changes during reductive heating c) Linear combination analysis of Ir and Os L_{III} XANES data within the Os-Ir bimetallic system. For Os, an intermediate is found during the initial reductive heating, which is then converted to the reduced final state.

Rh-Os bimetallic system

In the Rh-Os bimetallic system, the formation of one *hcp* phase and one *fcc* phase is observed (**Fig. S12**). The *fcc* phase has much sharper Bragg peaks indicating a significantly more crystalline phase than the *hcp* phase. The lattice parameter obtained from Rietveld refinement for the *fcc* phase (a=3.8147(7) Å) is in good agreement with the literature value[23] for Rh at 500 °C (a=3.8104 Å). Similarly, the hcp lattice is close to that of Os (a=2.7368 °C, c=4.3225 Å at 500 °C)[22]. Based on the slightly expanded fcc lattice compared to Rh and a slightly contracted *hcp* lattice with respect to pure Os, it cannot be excluded that minor contributions of Os in the Rh-dominated *fcc* phase occur and, vice versa, Rh in the predominantly osmium-based *hcp* phase.

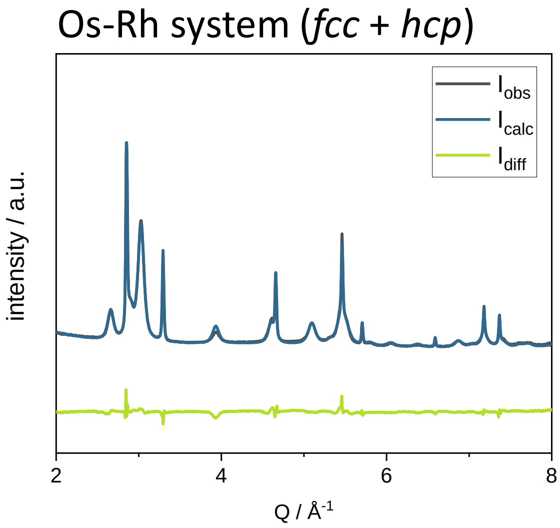

		R_{wp} = 9.5 %
fcc	*a* = 3.8147(7) Å	
	V = 55.51(2) Å^3	43.2(2) wt.%
hcp	*a* = 2.7292(1) Å	*c* = 4.3353(3) Å
	V = 27.97(2) Å^3	56.8(3) wt.%

Figure S12 Rietveld refinement with lattice parameters obtained for the final frame at 500 °C of the *in situ* study within the Os-Rh bimetallic system, for a two phasic system with one *fcc* phase (225, Fm-3m) and one *hcp* phase (194, $P6_3/mmc$).

The *in situ* study of the phase formation shows a crystalline precursor intermediate form alongside the metallic phases during reductive heating (**Fig. S13**). Analysis of the XANES data reveals similarly an intermediate during the Os reduction. This is, however, already visible at lower temperatures before the crystalline intermediate is observed. The combined *in situ* data suggest that osmium, at least partially, forms an intermediate that crystallizes before Os is reduced to the metallic state. This stepwise reduction process leads to a separation of Rh and Os in the system and a two-phase system is obtained.

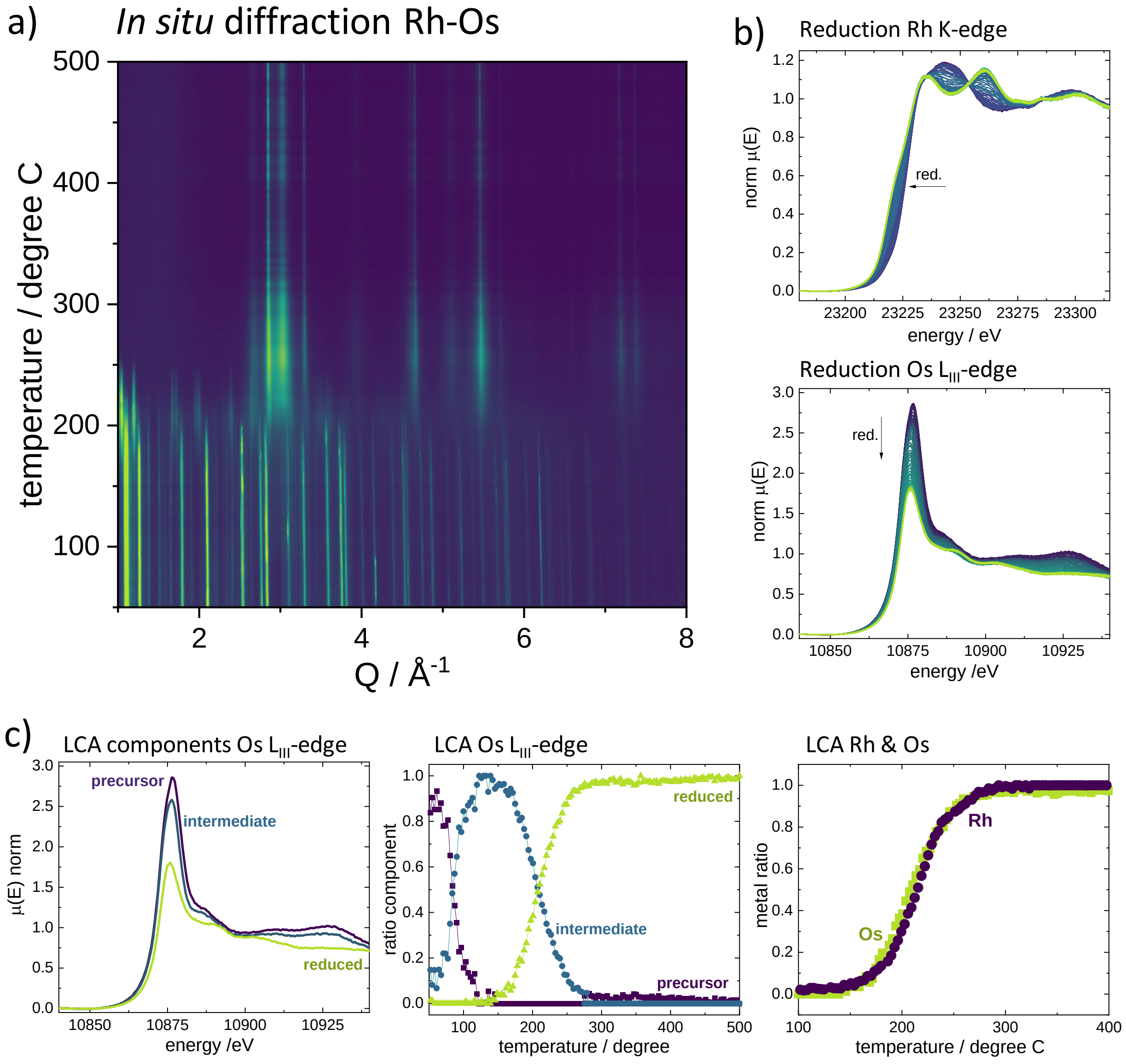

Figure S13 *In situ* XRD/XAS study of phase formation in the Os-Rh bimetallic system. a) Contour plot of the *in situ* X-ray diffraction data collected during reductive heating. b) X-ray absorption spectra (XANES) of the Rh K-edge and Os L_{III}-edge during the reductive heating to follow the reduction of the individual elements. Arrows indicate the changes during reductive heating c) Linear combination analysis of Rh K-edge and Os L_{III}-edge XANES data within the Os-Rh bimetallic system. For Os, an intermediate is found during the initial reductive heating, which is then converted to the reduced final state.

Ru-Pt bimetallic system

In the Ru-Pt bimetallic system, a phase mixture consisting of two *fcc* phases, as well as an *hcp* phase, is found (see **Fig. S14**). The *fcc* phase with a larger lattice parameter (3.94 Å) agrees with the lattice parameter of Pt[24] at 500 °C, which indicates the presence of circa 10% platinum particles. The *hcp* lattice parameters found are slightly larger than the ones of pure ruthenium (a = 2.71Å, c = 4.29 Å at 500 °C)[24], especially in the c-direction, indicating a partial contribution of Pt in the Ru lattice. The dominating *fcc* phase has a lattice constant a smaller than that of pure Pt, which agrees with the incorporation of Ru into the lattice and can be attributed to a Pt-Ru alloy.

Ru-Pt system (2 *fcc* + *hcp*)

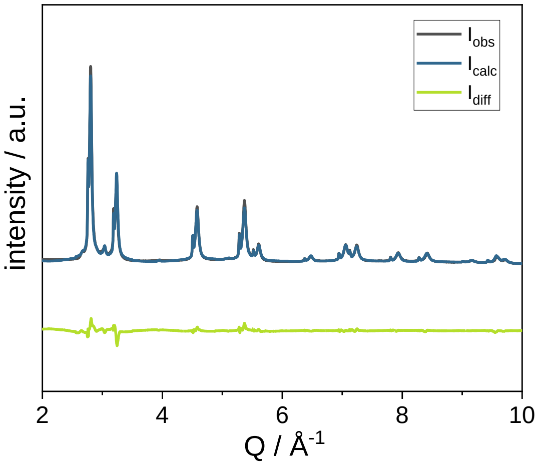

		R_{wp} = 13.2 %
fcc	a = 3.8807(8) Å	
	V = 82.1(5) Å^3	58.4(1) wt.%
fcc	a = 3.9418(8) Å	
	V = 61.2(1) Å^3	9.1(2) wt.%
hcp	a = 2.7182(4) Å	c = 4.3308(14) Å
	V = 27.71(1) Å^3	8.9(2) wt.%

Figure S14 Rietveld refinement of the with lattice parameters obtained for the final frame at 500 °C of the *in situ* study within the Ru-Pt bimetallic system, for a three phase mixture with two *fcc* phases (225, Fm-3m) and one *hcp* phase (194, $P6_3$/mmc).

The *in situ* study (**Fig. S15**) of the reduction of Ru and Pt shows a crystallization of the metallic phases at temperatures as low as 150 °C. The XANES analysis shows that platinum is reduced at temperatures lower than the reduction of ruthenium in the sample. Further, an intermediate phase crystallizes at temperatures agreeing with the precursor reduction (around 150 °C). This can be attributed to the crystallization of NH_4Cl (see **Fig. S15c**), which is a product of the precursor decomposition[16]. Conversion of the high-temperature polymorph of NH_4Cl can be followed (see **Fig. S15 a&c**), which is followed by sublimation during continuous reductive heating.

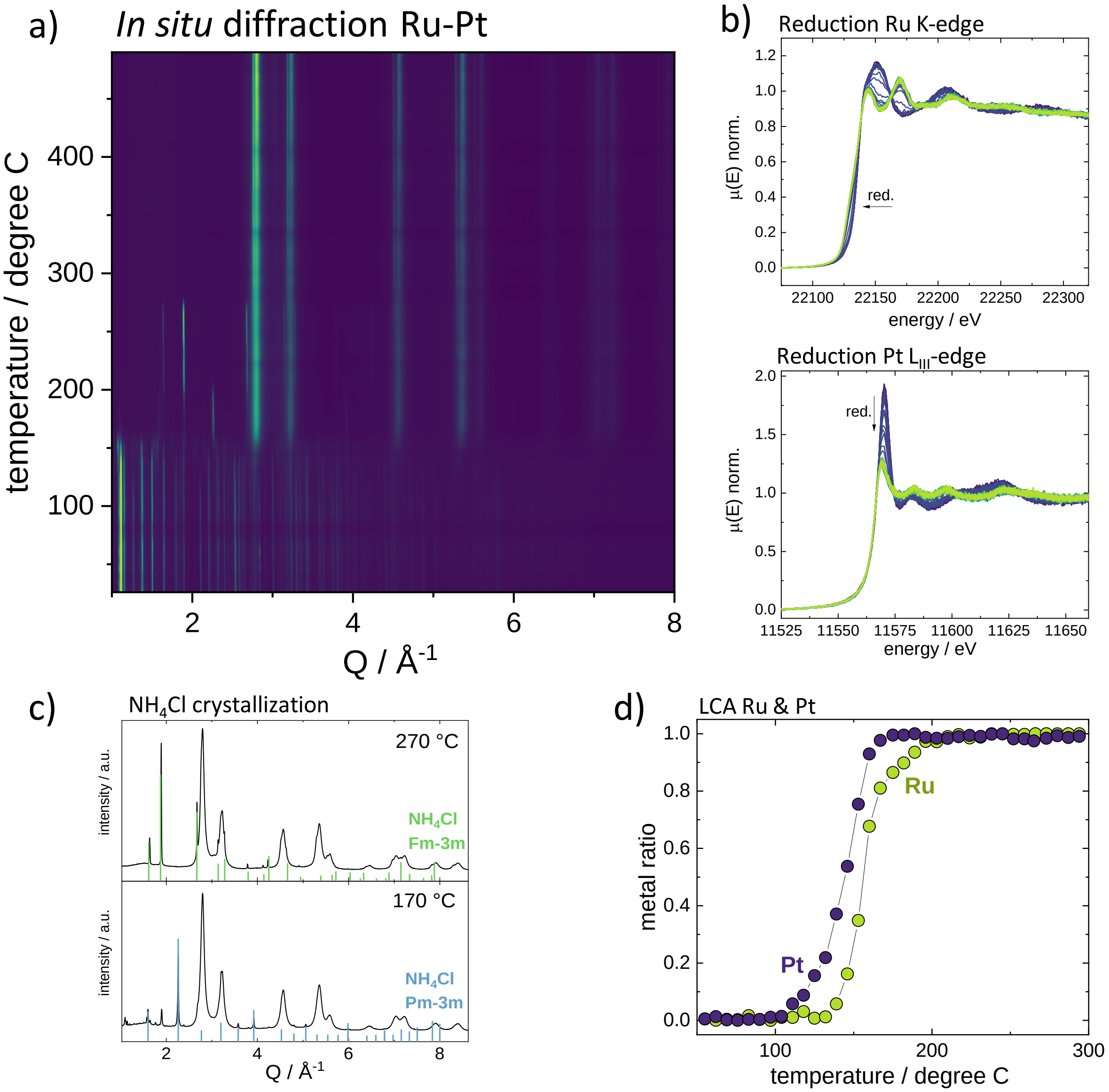

Figure S15 *In situ* XRD/XAS study of phase formation in the Ru-Pt bimetallic system. a) Contour plot of the *in situ* X-ray diffraction data collected during reductive heating. b) X-ray absorption spectra (XANES) of the Ru K-edge and Pt L_{III}-edge during the reductive heating to follow the reduction of the individual elements. Arrows indicate the changes during reductive heating c) Diffraction patterns collected at 170 °C and 270 °C showing the crystallization of NH_4Cl and its phase transformation to the high temperature polymorph d) Linear combination analysis results showing the ratio of metal present at respective temperatures determined from the Ru K-edge and Ir L_{III}-edge XANES data within the Ru-Pt bimetallic system.

Ru-Ir bimetallic system

In the bimetallic system of Ru and Ir, the formation of a two-phase mixture consisting of an *fcc* phase that contributes 25% and an *hcp* phase with 75% contribution is found (see **Fig. S16**). The lattice parameter determined for the *fcc* phase is in good agreement with the literature value of Ir at 500 °C (*a* = 3.84 Å)[21]. The lattice parameters determined for the hcp phase are slightly larger than the literature values for pure ruthenium at 500 °C (*a* = 2.71Å, *c* = 4.29Å)[25]. Based on the 50/50 composition of the Ir-Ru system as determined by EDX analysis (see **Fig. S4**), this agrees well with the crystallization of a pure iridium fcc phase as well as a mixed Ir-Ru phase that crystallizes in an *hcp* lattice. It has to be noted that the Rietveld refinement poorly described the relative heights of Bragg peaks in the *hcp* phase, which has been observed also in e.g. the HEA sample crystallizing in an *hcp* lattice. This can be ascribed to a very defective structure of the particles formed (see discussion in the main text).

In the *in situ* diffraction study (see **Fig. S17**), no initial crystallization of iridium can be made out. The XANES data analysis, however, reveals that iridium reduces at temperatures slightly lower than the reduction of ruthenium, which agrees with a spatial separation of iridium and ruthenium in the sample. Furthermore, the crystallization and following sublimation of NH_4Cl is found, as discussed for the Ru-Pt system (see **Fig. S15** and corresponding text.)

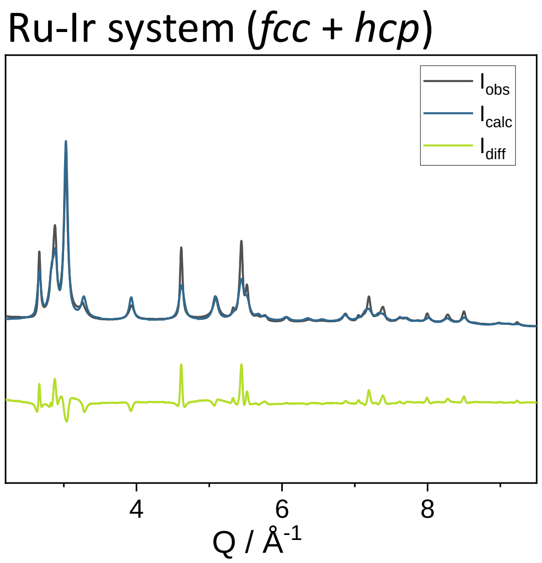

		R_{wp} = 25.0 %
fcc	*a* = 3.8361(4) Å	
	V = 56.5(1) Å³	24.8(4) wt.%
hcp	*a* = 2.7236(3) Å	*c* = 4.3548(5) Å
	V = 27.98(1) Å³	75.2(6) wt.%

Figure S16 Rietveld refinement of the with lattice parameters obtained for the final frame at 500 °C of the *in situ* study within the Ru-Ir bimetallic system, for a two phase mixture with one *fcc* phase (225, Fm-3m) and one *hcp* phase (194, $P6_3/mmc$).

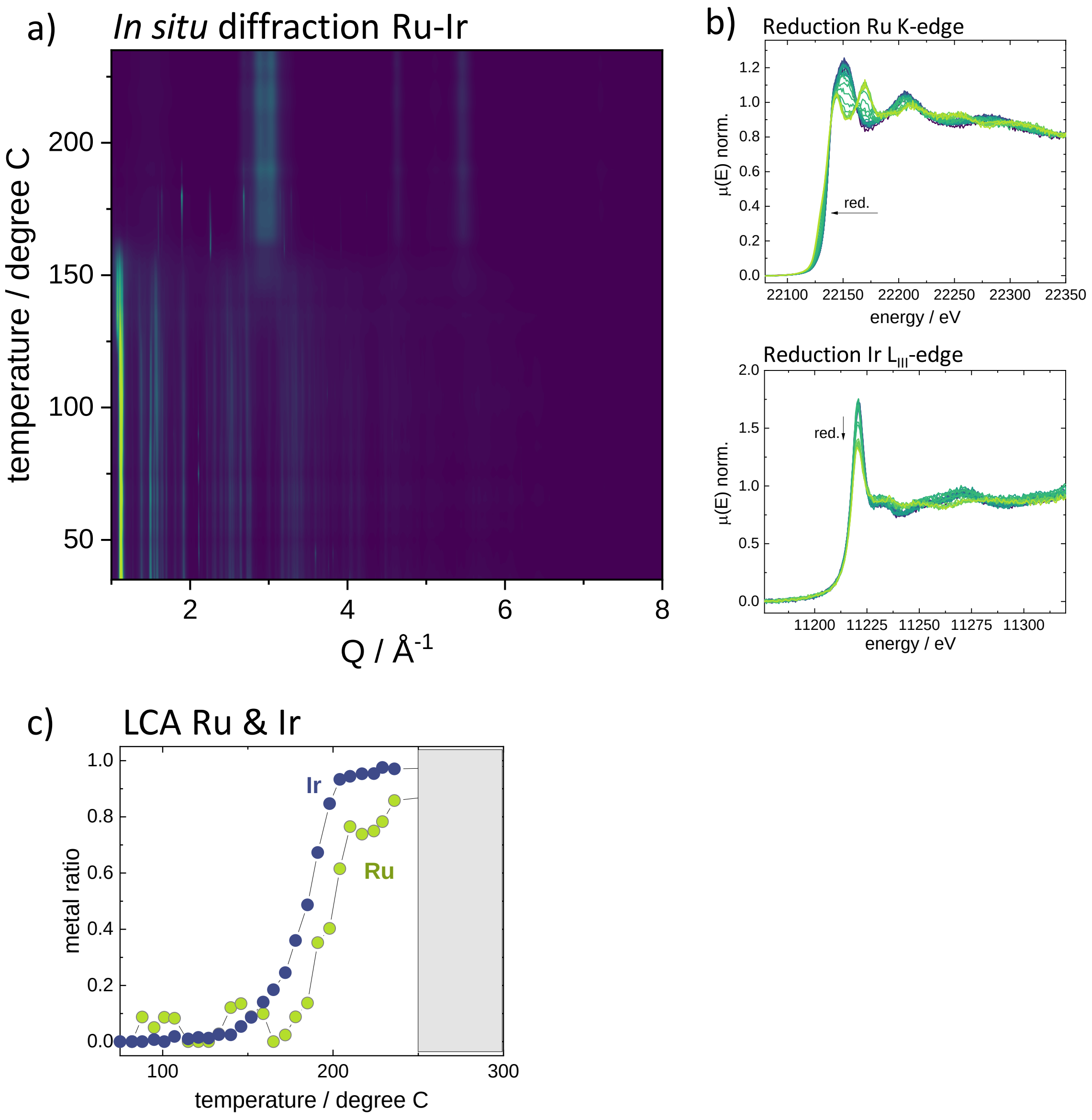

Figure S17 *In situ* XRD/XAS study of phase formation in the Ru-Ir bimetallic system. a) Contour plot of the *in situ* X-ray diffraction data collected during reductive heating. b) X-ray absorption spectra (XANES) of the Ru K-edge and Ir L_{III}-edge during the reductive heating to follow the reduction of the individual elements. Arrows indicate the changes during reductive heating c) Linear combination analysis, fit of the ratio of metal determined from the Ru K-edge and Ir L_{III}-edge XANES data within the Ru-Ir bimetallic system

Ir-Pt bimetallic system

In the Ir-Pt bimetallic system formation of three distinguishable *fcc* phases is observed (see **Fig. S18**), which can be attributed to the formation of both an Ir-phase (3.83 Å, 15%) and a Pt-phase (3.92 Å, 20%), where the lattice parameters agree well with the literature values of both Ir[21] and Pt[24]. A major contribution is found for a mixed Ir-Pt alloy phase (3.87 Å, 65%).

The *in situ* diffraction data (see **Fig. S19**) clearly show that an initial *fcc* phase, with a lattice that agrees with the lattice parameter of Platinum, starts to crystallize at temperatures as low as 150 °C. This agrees well with the X-ray absorption spectroscopy data where an initial reduction of platinum is found starting at temperatures as low as 125 °C. About 50% of the platinum present in the sample is reduced at 150 °C, while the remaining platinum is reduced at higher temperatures, which goes alongside the reduction of iridium in the sample. This step-wise reduction of platinum explains the formation of separate platinum and iridium samples as well as a mixed Ir-Pt alloy.

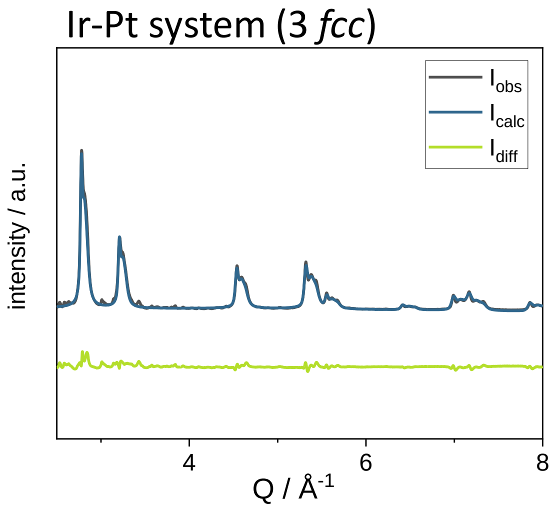

		R_{wp} = 14.4 %
fcc - 1	*a* = 3.8316(6) Å	
	V = 56.25(2) $Å^3$	15.2(2) wt.%
fcc - 2	*a* = 3.8748(5) Å	
	V = 58.18(1) $Å^3$	64.8(11) wt.%
fcc - 3	*a* = 3.9175(2) Å	
	V = 60.12(1) $Å^3$	20.1(8) wt.%

Figure S18 Rietveld refinement results with lattice parameters obtained for the final frame at 250 °C of the *in situ* study within the Ir-Pt bimetallic system, for a three phase mixture with three *fcc* phases (225, Fm-3m).

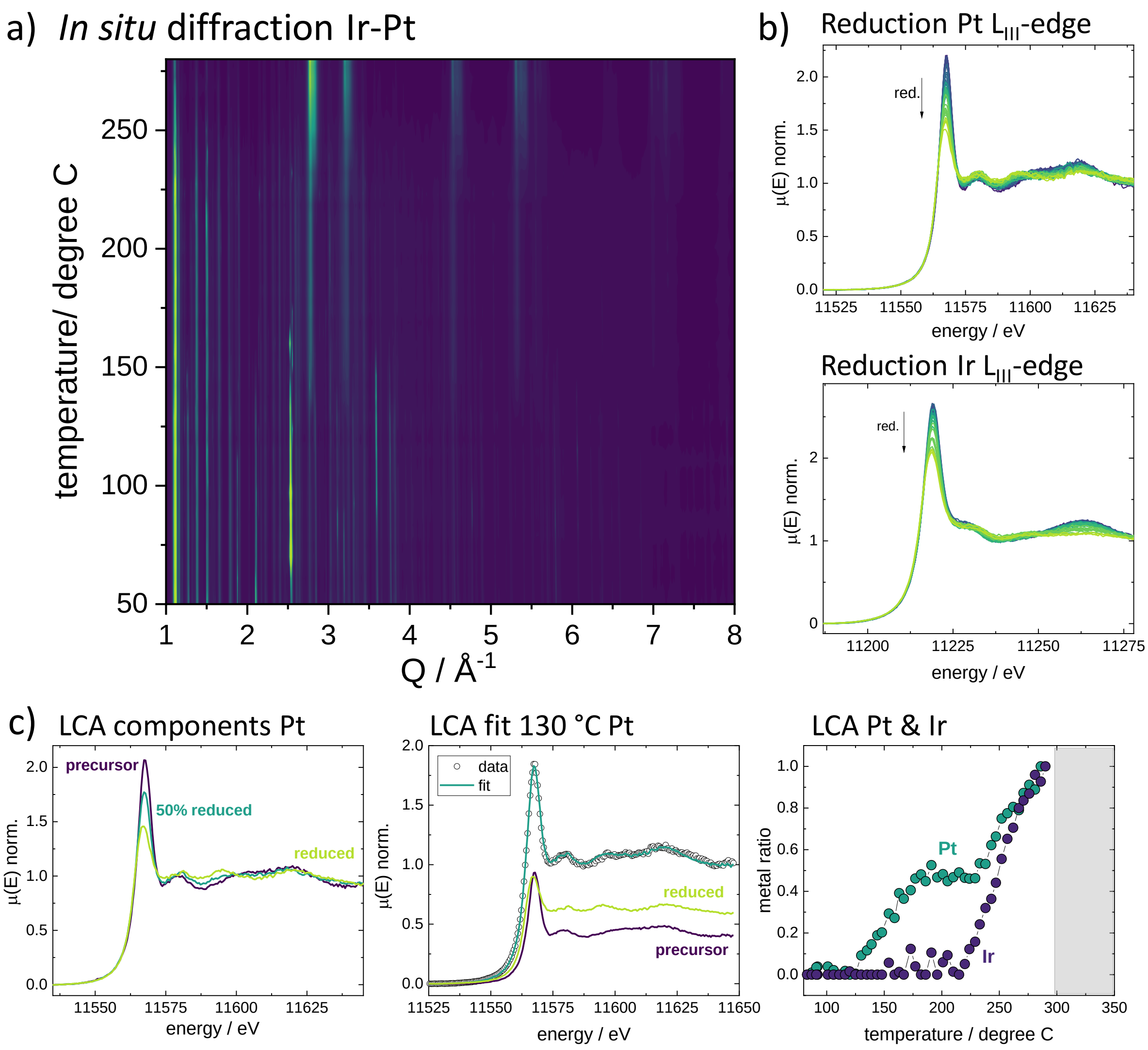

Figure S19 *In situ* XRD/XAS study of phase formation in the Ir-Pt bimetallic system. a) Contour plot of the *in situ* X-ray diffraction data collected during reductive heating. b) X-ray absorption spectra (XANES) of the Ir and Pt L_{III}-edges during the reductive heating to follow the reduction of the individual elements. Arrows indicate the changes during reductive heating c) Linear combination analysis of Pt and Ir L_{III}-edge XANES data within the Ir-Pt bimetallic system. For Pt, an initial reduction of 50% of the present platinum is observed in a temperature range around 150-200 °C of the reductive heating with the residual Pt being reduced at higher temperatures. The grey area signifies missing data due to a temporary loss of X-ray beam.

Studies of five-metal HEA nanoparticles

<u>In situ studies of *fcc*-HEA and *hcp*-HEA formation</u>

XRD patterns (individual frames) from in situ studies

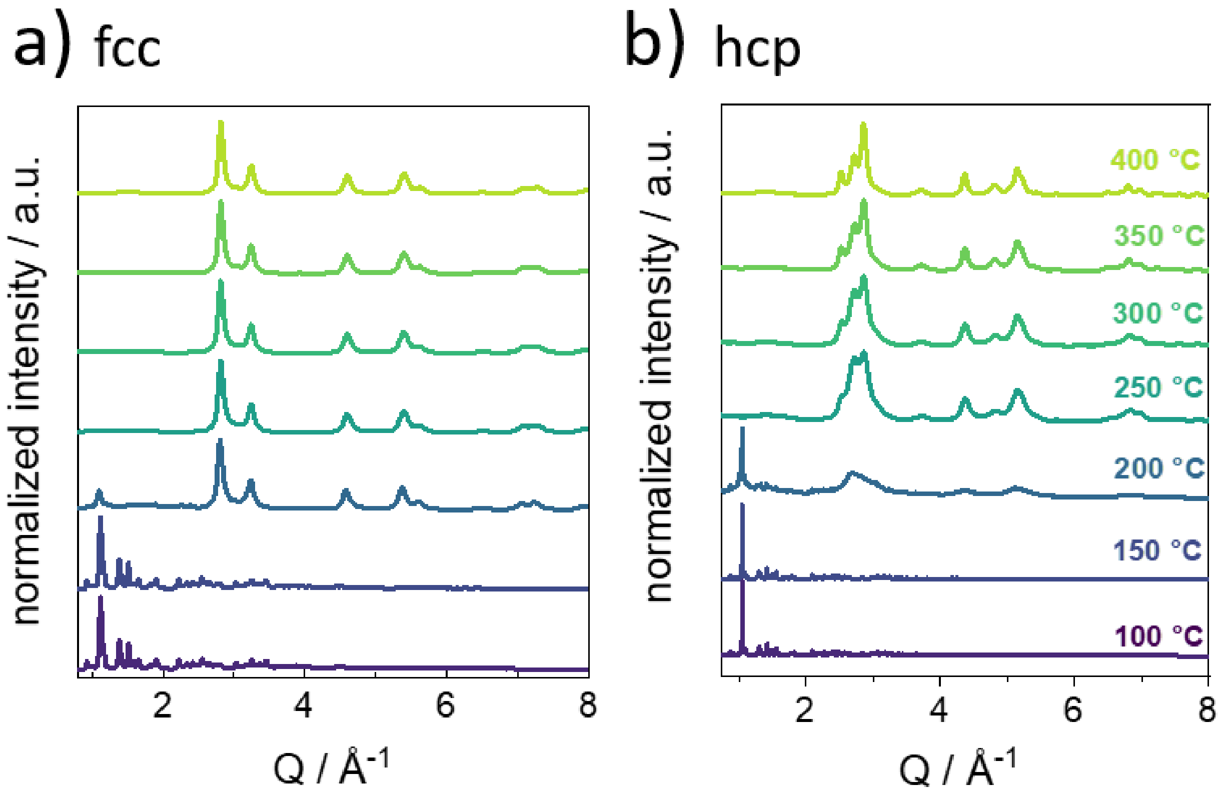

Figure S20 X-ray diffraction patterns taken at different temperatures during the reductive heating showing the gradual precursor reduction and crystallization of metallic particles a) in the *fcc*-HEA and b) in *the* hcp-HEA system.

Contour plots of fcc-HEA in situ XANES spectra

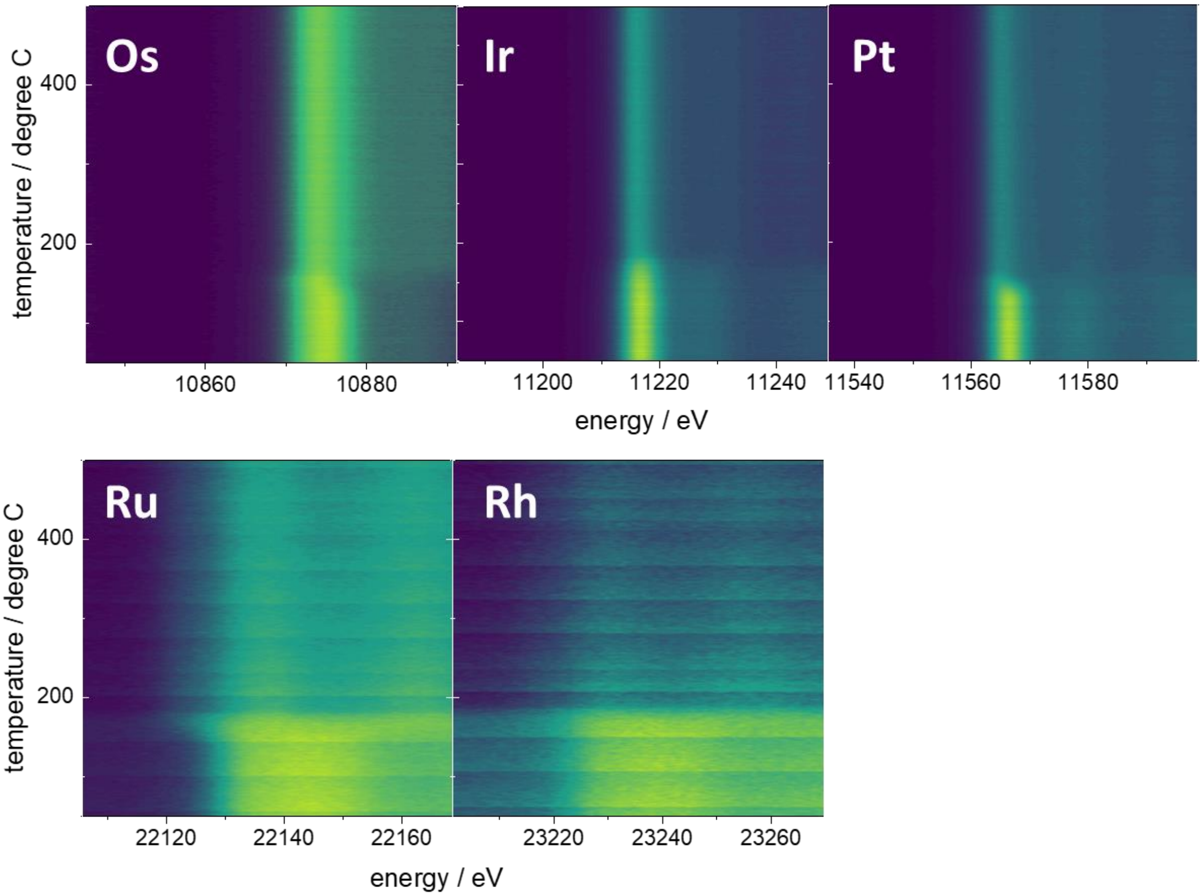

Fig S21. Contour plots of in *situ* XANES spectra collected during the heat treatment in H_2/He gas stream of the *fcc*-HEA sample, XANES spectra were continuously collected in fluorescence mode at the L_{III}-edges (Os, Ir, Pt) and the K-edges (Rh, Ru) while the sample was heated with a heat ramp of 100 K/h.

Sequential Rietveld refinement – fcc-HEA

A detailed analysis of the sequential Rietveld refinement of the *in situ* diffraction data collected for the *fcc*-HEA sample reveals that the lattice contraction of the formed *fcc* phase can be correlated with the reaction progress. An initial steep contraction is observed which levels off at around 220 °C. This agrees with a complete reduction of the metals present in the single-source precursor, as determined from the XANES analysis (indicated by a thick grey line in **Fig S22a**.) A second pronounced lattice contraction step occurs that can be aligned with the reaction progress of forming the *fcc*-phase, as determined from the scale factor. The maximum scale factor, which corresponds to a complete crystallization of the metals present in the *fcc* phase, is found at 240 °C and is marked with a thin light-grey line in **Fig S22a**. In the following heat treatment up to circa 320 °C, the lattice parameter remains largely constant and starts to contract even further when the heating ramp reaches temperatures above 350 °C. The coherent scattering domain size determined from the peak profile of the Bragg peaks shows that the domains grow during the heat treatment, as expected. The most pronounced crystallite growth is observed for heating above 350 °C. At lower temperatures, the domain size increases only slightly while the lattice strain, on the other hand, increases much more pronounced. The increase in maximum strain largely correlates with the decrease in unit cell parameter, showing that the formation of the high entropy alloy lattice with successive incorporation of five different elements leads to a very strained *fcc* crystal lattice. Maximum strain is found at a heating temperature of circa 375 °C. At higher temperatures, the strain decreases slightly again, in what is probably a lattice relaxation and crystal growth step. There is, however, still a significant amount of lattice strain found for the larger crystal domains that are obtained after heating to 500 °C.

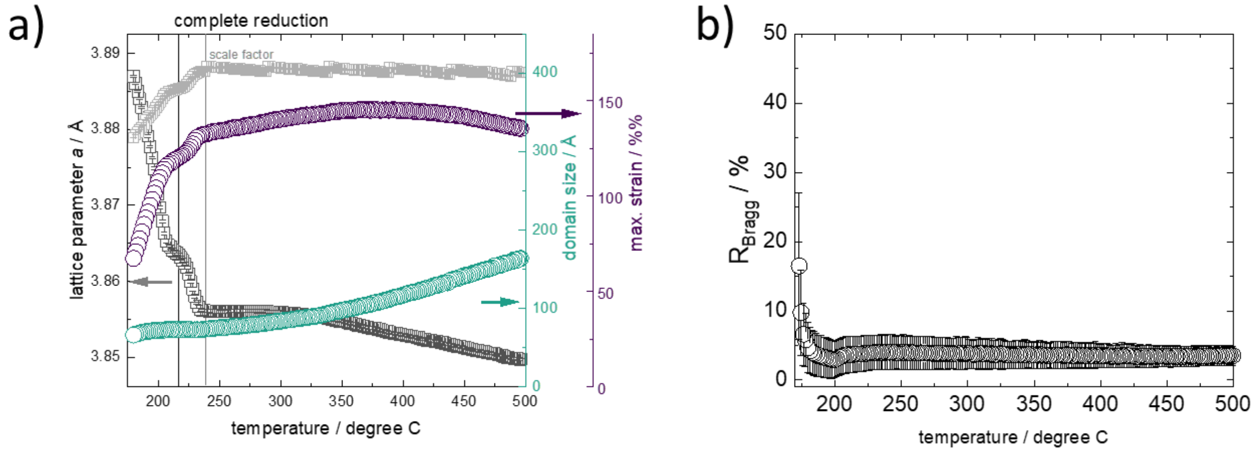

Figure S22 a) Evolution of lattice parameter a, apparent domain size, and maximum lattice strain, as well as the scale factor obtained for the sequential Rietveld refinement of the *in situ* diffraction data collected for the *fcc*-HEA phase and b) evolution of R_{Bragg} values of the sequential refinement as a function of the heating temperature.

Contour plots of hcp-HEA in situ XANES spectra

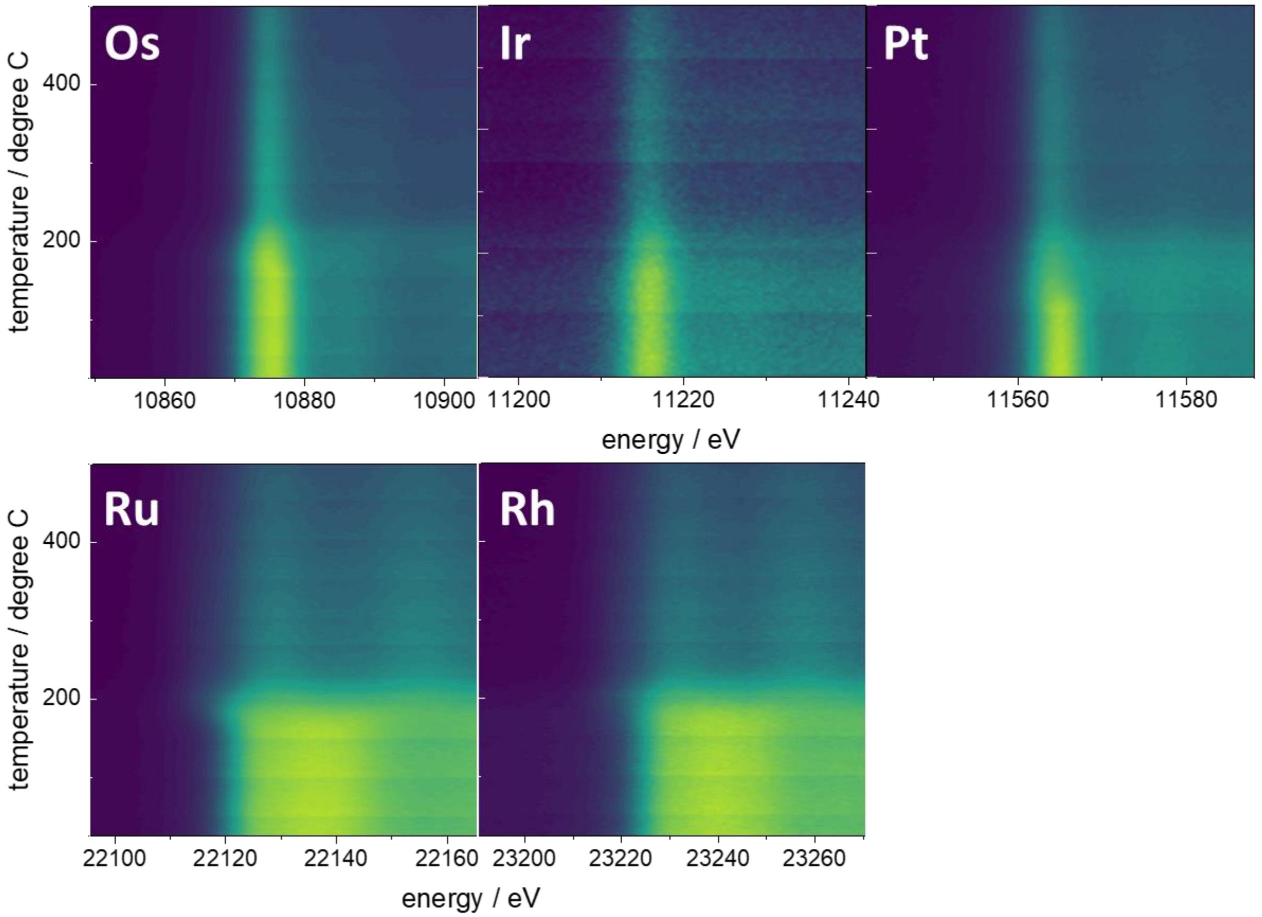

Figure S23 Contour plots of *in situ* XANES spectra collected during the heat treatment in H_2/He gas stream of the *hcp*-HEA sample, XANES spectra were continuously collected in fluorescence mode at the L_{III}-edges (Os, Ir, Pt) and the K-edges (Rh, Ru) while the sample was heated with a heat ramp of 100 K/h.

Sequential Rietveld refinement – hcp-HEA

The quality of the sequential Rietveld refinement of the *in situ* diffraction data collected from the *hcp* HEA sample is limited due to the very broad Bragg peaks observed. The relative peak heights of individual Bragg peaks further do not correspond to the expected peak ratios for a crystalline bulk *hcp* phase. This could indicate the presence of a significant number of defects, such as stacking faults. A slight improvement can be achieved when adding an *fcc* phase to the model, to represent stacking faults in the structure (see also HR-TEM, **Fig. S30**). The presence of individual particles of *fcc* structure is however unlikely, as the (200) reflection, the only high-intensity Bragg reflection that does not overlap with Bragg peaks from the *hcp* phase, is absent in the data (see **Fig. S24**). The *fcc*-phase is however helpful in describing the Bragg peaks at 2.9, 4.6, and 5.4 Å^{-1} ((002), (110), and (112) in the *hcp* phase), which are significantly more dominant at lower temperatures in the formation mechanism.

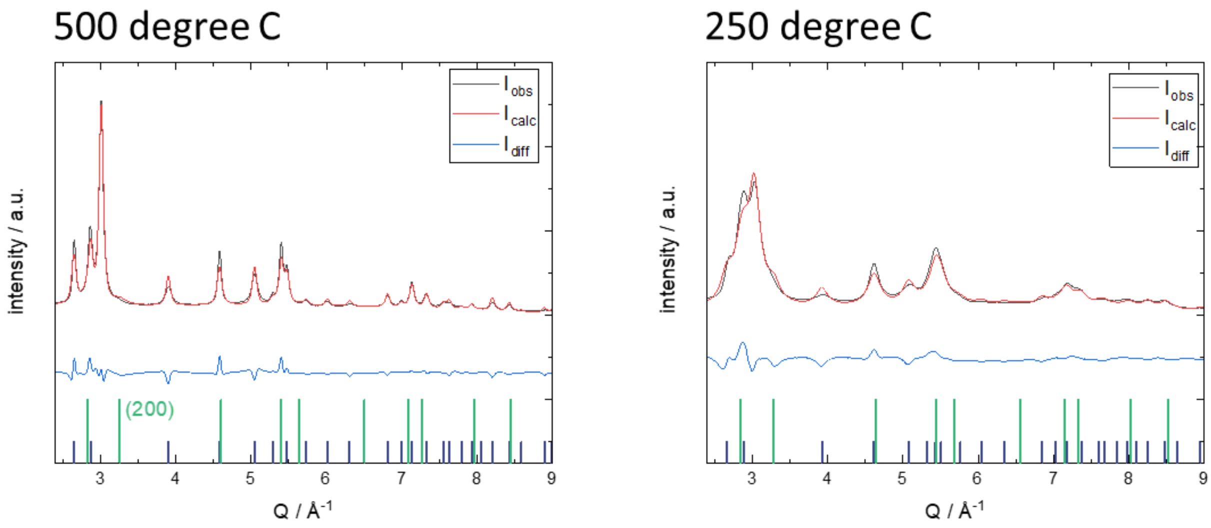

Figure S24 Rietveld refinement of X-ray diffraction pattern at 500 degrees C and 250 degrees C, XRD was collected of the *hcp*-HEA sample during the reductive heating. A two-phase model with one *hcp* and one *fcc* phase was used to fit the data.

In the sequential refinement, the lattice parameter of the added *fcc* phase was kept constant at *a* = 3.83 Å, while the *hcp*-phase lattice parameters *a* and *c* were refined freely. The weight fraction of the hcp phase increases constantly during the thermolysis, as well as the refined crystallite domain size of the *hcp* phase (see **Fig. S24a**). The crystallite size of the added *fcc* phase remains rather constant, with only a small size increase during the heating protocol. The loss of *fcc*-character of the diffraction data can for example be due to a loss of *fcc*-like atomic arrangements while the hcp phase crystallizes and, and *fcc*-like stacking faults or *fcc*-phase domains become less frequent. The large amount of *fcc*-phase in the initial refinements could also be due to very broad Bragg peaks in the initial crystallization stages at low synthesis temperatures, where the individual Bragg peaks of the hcp phase cannot be distinguished and the *fcc* phase can describe these very broad diffraction

peaks equally well. Due to the similarity of the atomic arrangements of both *fcc* and *hcp* phases, it cannot be clearly distinguished if the crystallization of the *hcp*-HEA phase proceeds via an initially formed *fcc* phase. The refined strain remains constant for both the *fcc* and *hcp* phases (25%% and 7%%, respectively). The peak broadening in the *hcp*-HEA sample seems, thus, dominated by small domain sizes - with defects, grain boundaries, and stacking faults - rather than a very strained lattice. During the heat treatment, the *hcp* lattice parameters *a* and *c* both increase (**Fig. S24c**), which leads to an overall increased unit cell volume.

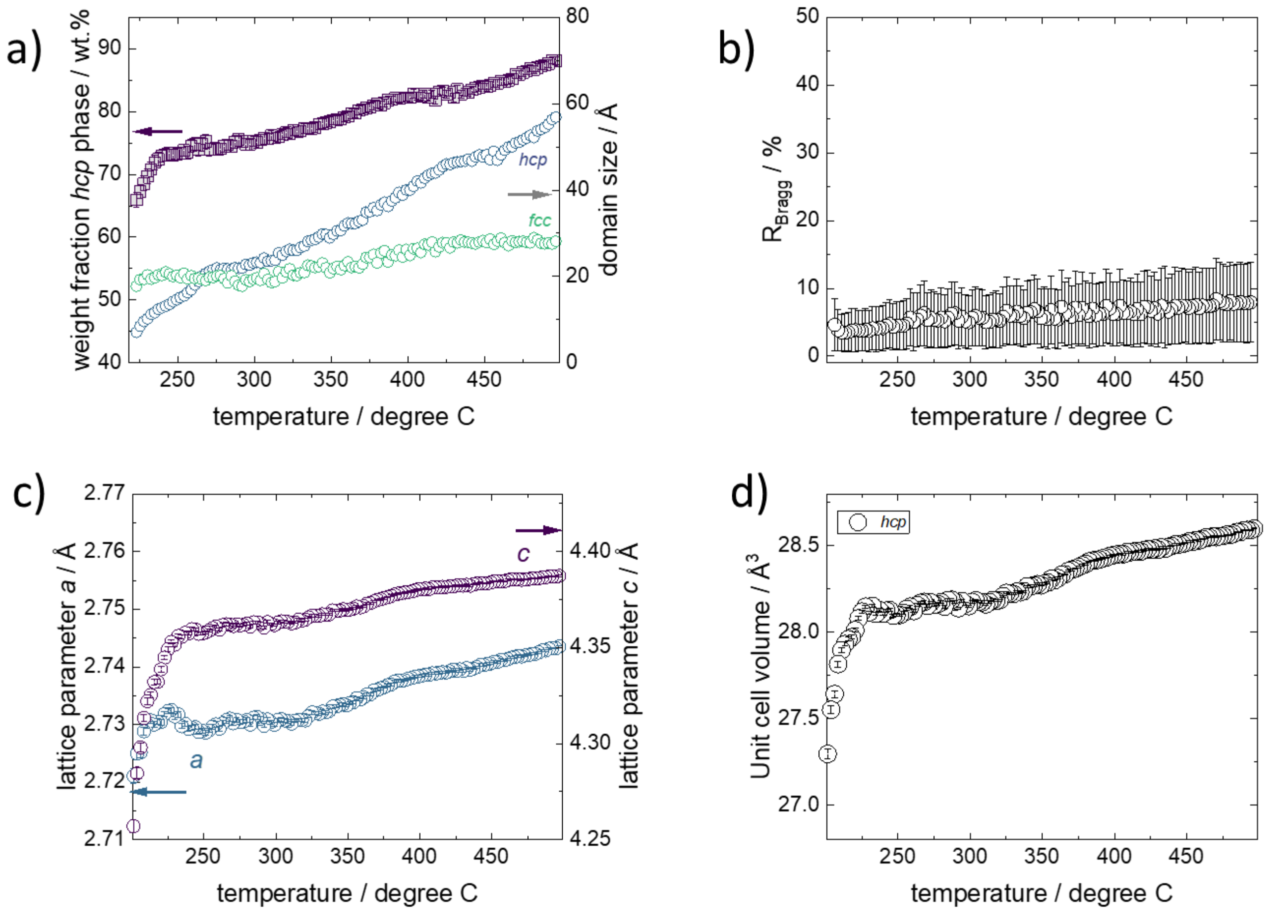

Figure S25 Variables determined by sequential Rietveld refinement of the *in situ* diffraction data collected for the *hcp*-HEA sample showing as a function of the heating temperature a) the weight fraction and apparent domain size of both hcp and fcc phase, b) the R_{Bragg} values of the hcp phase, c) the lattice parameters a and c of the *hcp* phase as well as the corresponding d) unit cell volume. The *fcc* lattice was kept constant during the refinement.

Ex situ characterization of HEA samples

EDX analysis HEA samples

Table S3. Elemental composition of the HEA nanoparticle powder samples (*fcc*-HEA and *hcp*-HEA) as determined by Energy-dispersive X-ray spectroscopy (EDX).

Fcc-HEA

Statistic	Ru	Rh	Os	Ir	Pt
Max	11.84	28.30	7.02	23.33	38.22
Min	9.01	22.41	6.20	20.56	32.15
Mean	***10.65***	***26.28***	***6.49***	***21.66***	***34.92***
Standard deviation	1.04	2.33	0.32	1.03	2.19

Hcp-HEA

Statistic	Ru	Rh	Os	Ir	Pt
Max	33.05	9.39	32.87	16.49	16.35
Min	27.40	7.59	26.50	13.37	14.22
Mean	***31.35***	***8.72***	***29.81***	***14.68***	***15.33***
Standard deviation	1.97	0.54	1.83	1.01	0.76

XRD and PDF of *hcp*-HEA sample

Similar to the Rietveld refinement of the *in situ* diffraction dataset, the quality of the real-space refinement of PDFs for the *hcp*-HEA sample could be improved, when an *fcc* phase was added to the model (see **Figure S26**, and **Table S16**). For the *fcc*-phase, a very small apparent crystallite size of 26 Å was obtained, while for the hcp-phase the crystallite size of 70 Å was significantly larger. These results are in line with what was observed in the *in situ* data set. The improvement of fit with including an *fcc* structure points toward the presence of either a significant amount of stacking faults, which can be described with an *fcc* structure, or to the presence of very small crystallite domains of a separate *fcc*-phase. To analyze whether an *fcc* content or corresponding stacking faults are present in the *hcp*-HEA sample, the total scattering data were also fit both with a single-phase *hcp* model and a two-phase *hcp* + *fcc* model. Also in the real-space refinement of the PDFs a significant improvement of the fit quality can be achieved when adding an *fcc* phase. This is most dominant at low r-ranges of around 20-25 Å. It has to be noted that the added *fcc*-phase could not be freely refined, but all parameters had to be pre-set, as otherwise unnaturally large lattice constants of > 5 Å were obtained. The parameters for the *fcc*-phase model were chosen based on the results obtained for the *fcc*-phase contribution determined by Rietveld refinement of the diffraction data, these correspond to the atom atom distances also obtained in the hcp phase.

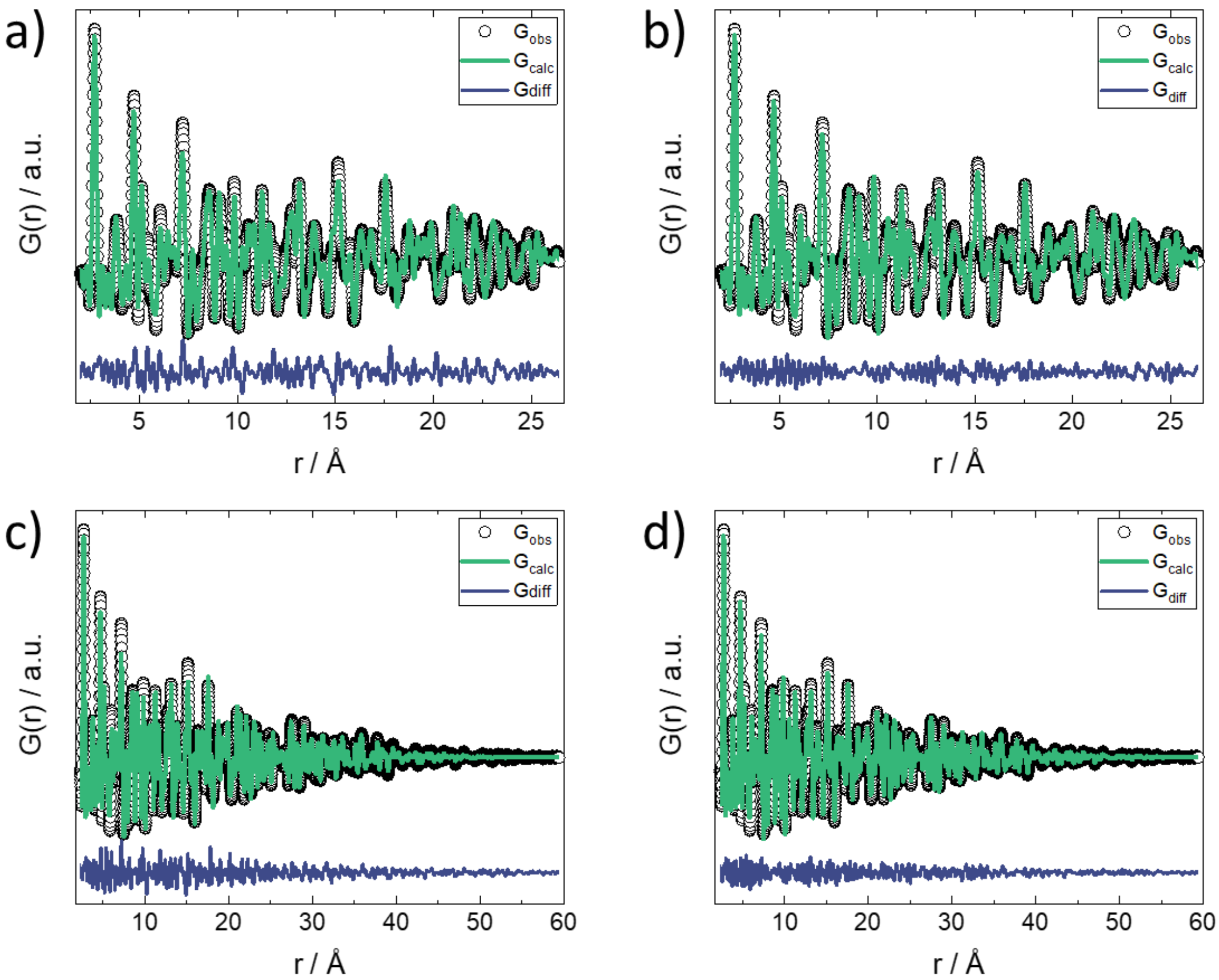

Figure S26 PDF of the ex-situ hcp-HEA sample fit with a single *hcp* phase (Rw=0.234) [a) and c)] and b) both an *hcp* and an *fcc* phase (R_w=0.165) [b) and d)]. The top panel, a) and b), show a magnification of the low-r region up to 25 Å, where the effect of adding an *fcc* phase has the largest contribution to improving the fit quality.

HRTEM and STEM-EDX of hcp-HEA nanoparticles

The atomic structure of the hcp-HEA particles is also investigated by scanning transmission electron microscopy combined with energy-dispersive X-ray spectroscopy (STEM-EDX). The elemental distribution maps created from STEM-EDX measurements for the *hcp*-phase HEA (**Fig. S27**) show the presence of all five-element, evenly distributed throughout the particles. The contrast variations that are clearly visible in the HR-TEM micrograph in **Figure S28** show pronounced planar defects that indicate significant stacking disorder in the *hcp*-HEA sample.

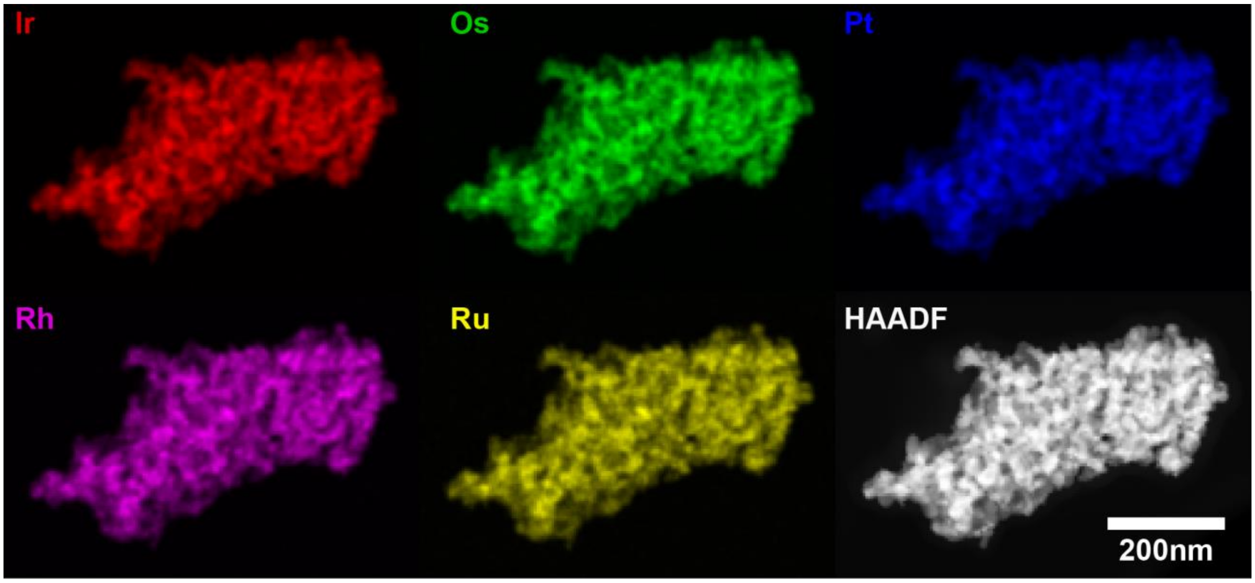

Figure S27 Element distribution in the particles of the *hcp*-HEA is visualized for individual metals by scanning transmission electron microscopy with energy-dispersive X-ray spectroscopy (STEM-EDX) and high-angle annular dark-field (HAADF) micrograph.

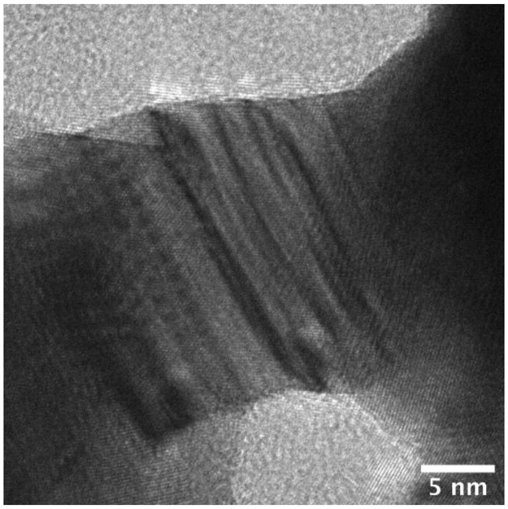

Figure S28 High-resolution transmission electron microscopy image of the *hcp*-HEA sample detailing the presence of stacking faults.

NMF analysis

Non-negative matrix factorization (NMF) was performed on selected STEM-EDX datasets using the Scikit-Learn[26] based implementation of the algorithm in HyperSpy. Based on inspection of a scree plot obtained from performing a Single value decomposition (implemented as PCA in HyperSpy) the output dimensions of the NMF decomposition were chosen to be four. This resulted in one background component containing mainly signal from the grid the three additional components presented and discussed for the *fcc* sample in the paper in **Figure 3.**

For the *hcp* –HEA particles, the second component consists mostly of carbon contaminations while the third component does not contain information.

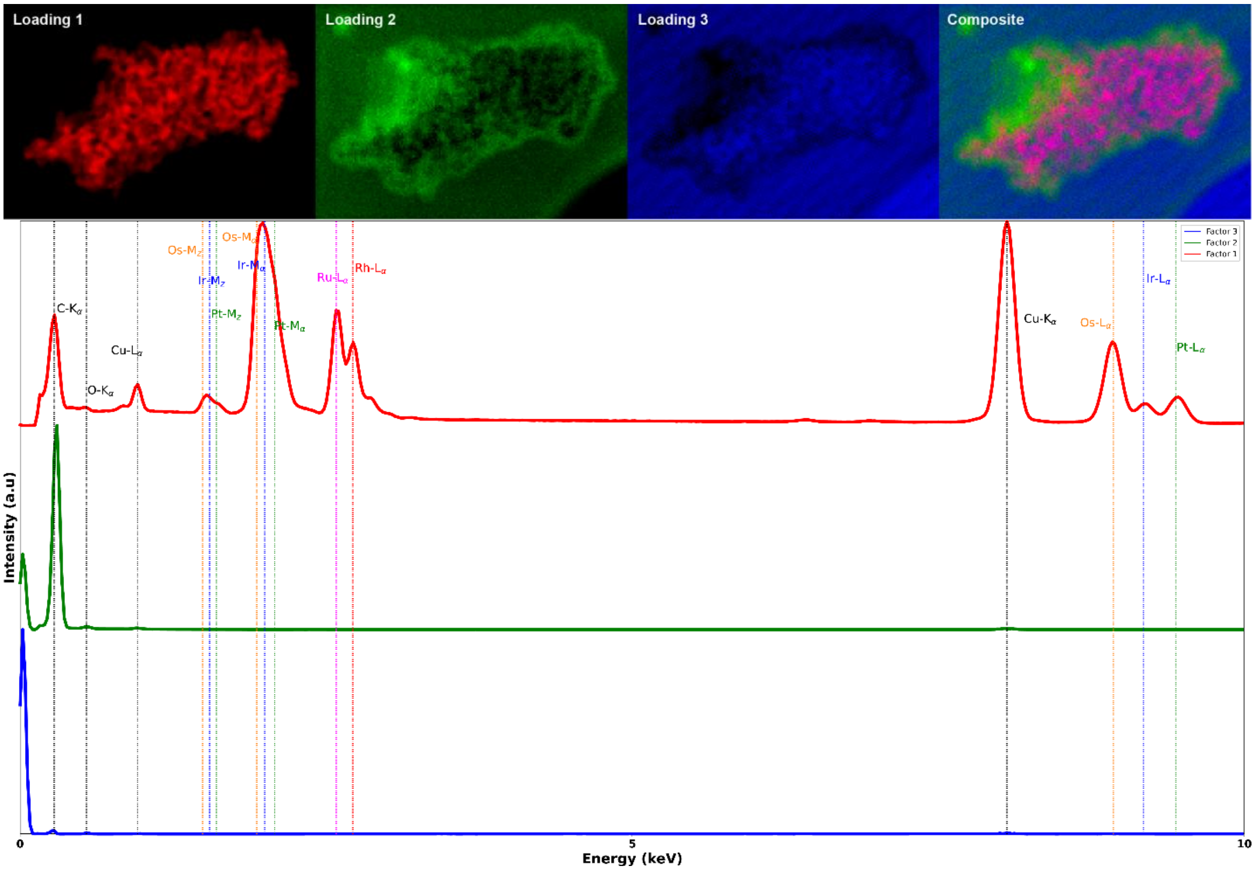

Figure S29 Individual loadings picked up by non-negative matrix factorization (NMF) with the corresponding composition of the individual factors that were identified by NMF analysis of STEM-EDX datasets collected for the *hcp*-HEA sample. Only Factor 1 contains contributions from the noble metals distributed in the HEA.

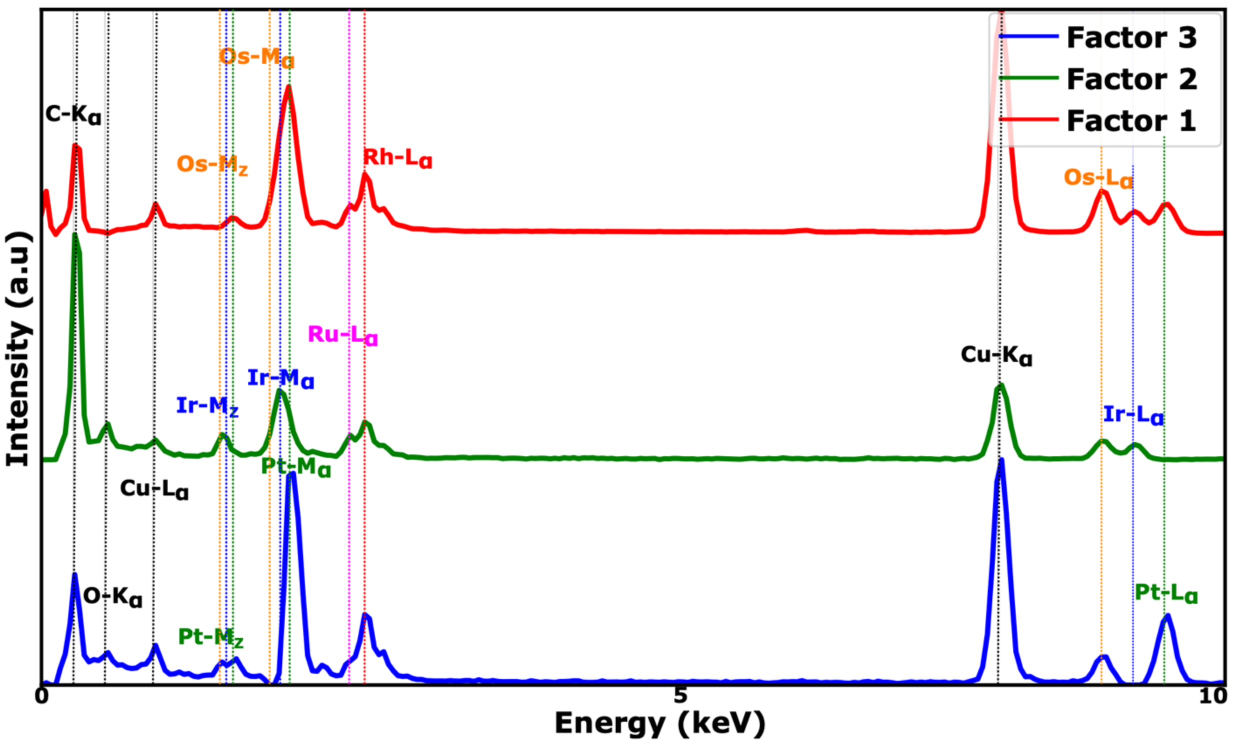

Figure S30 Composition of the individual factors that were identified by the non-negative matrix factorization of STEM-EDX datasets collected for the *fcc*-HEA sample.

Simulations

A simple simulation model was set up to visualize the effect of adding multiple elements on particle growth with as few assumptions as possible. The simulations only include the identity of the elements but no unique inter-element interactions, reducing the interactions to either homogeneous or heterogeneous bonds, respectively. Nanoparticle formation was simulated starting from a 13-atom core in a grid-based *fcc* structure, adding one atom to a randomly picked position on the surface of the core. Both the initial core atoms and the individual added atoms were randomized. All atoms were drawn from a pool of available atoms with an equal number of each element to ensure equimolar particle composition. Two adjustable parameters, apart from the number of distinct elements, were included in the simulation. Upon adding to the particle, the newly added atom is allowed to jump to adjacent sites for an adjustable number of iterations. This mimics kinetic energy for the atom to find the most stable configuration on the surface before losing the kinetic energy due to translational, rotational, and vibrational modes.

A score of 1.0 was "awarded" for each bond between the new atom and atoms present in the particle to evaluate the stability of the atomic position. This avoids the formation of under-coordinated sites, and thus reduces the surface energy as expected for a particle formation process. Additionally, heterogeneous bonds were modified by an adjustable score that represents e.g. mixing of elements with favorable mixing enthalpy by increasing the scores of the heterogeneous bonds. Conversely, a segregation-prone mixture was simulated by reducing the bond score of the heterogeneous bonds. Each atomic position visited by the new atom was thus evaluated according to the above rules and the position with the highest score was selected as the final position of the newly-added atom. New atoms were added up to a final particle size of 250 atoms.

To quantify the stochastic composition of a particle, the number of times each unique bond occurred was compared to the expected number of occurrences if the two atoms forming every bond were chosen randomly with equal probability:

$$E = (1 + \delta) N_{bonds} (\frac{1}{N_{elem}})^2$$

With N_{bonds} and N_{elem} being the number of bonds in the particle and the number of elements, respectively. δ is set to 0 for homogeneous bonds and 1 for heterogeneous bonds.

For this comparison Pearson's chi-squared was applied:

$$\chi^2 = \sum_{i}^{N_{unique}} \frac{(O_i - E_i)^2}{E_i}$$

with N_{unique} summing over each unique bond and calculated as $\frac{(2+N_{elem}-1)!}{2(N_{elem}-1)!}$.

O_i and E_i are the observed and expected number of bond i, respectively. This test was subsequently bootstrapped 500 times and the median χ^2-value was compared to the distribution of χ^2-values for 10000 randomly configured particles with the same number of elements. This yielded a p-value as the fraction of random particles with a higher χ^2-value than the median value which was used to construct the heatmaps in **Figure 4e** of the main paper. χ^2-distributions for a set of parameters (2 jumps and -0.50 score for heterogeneous bonds) are pictured in **Figure S30-S33** for two to five different elements.

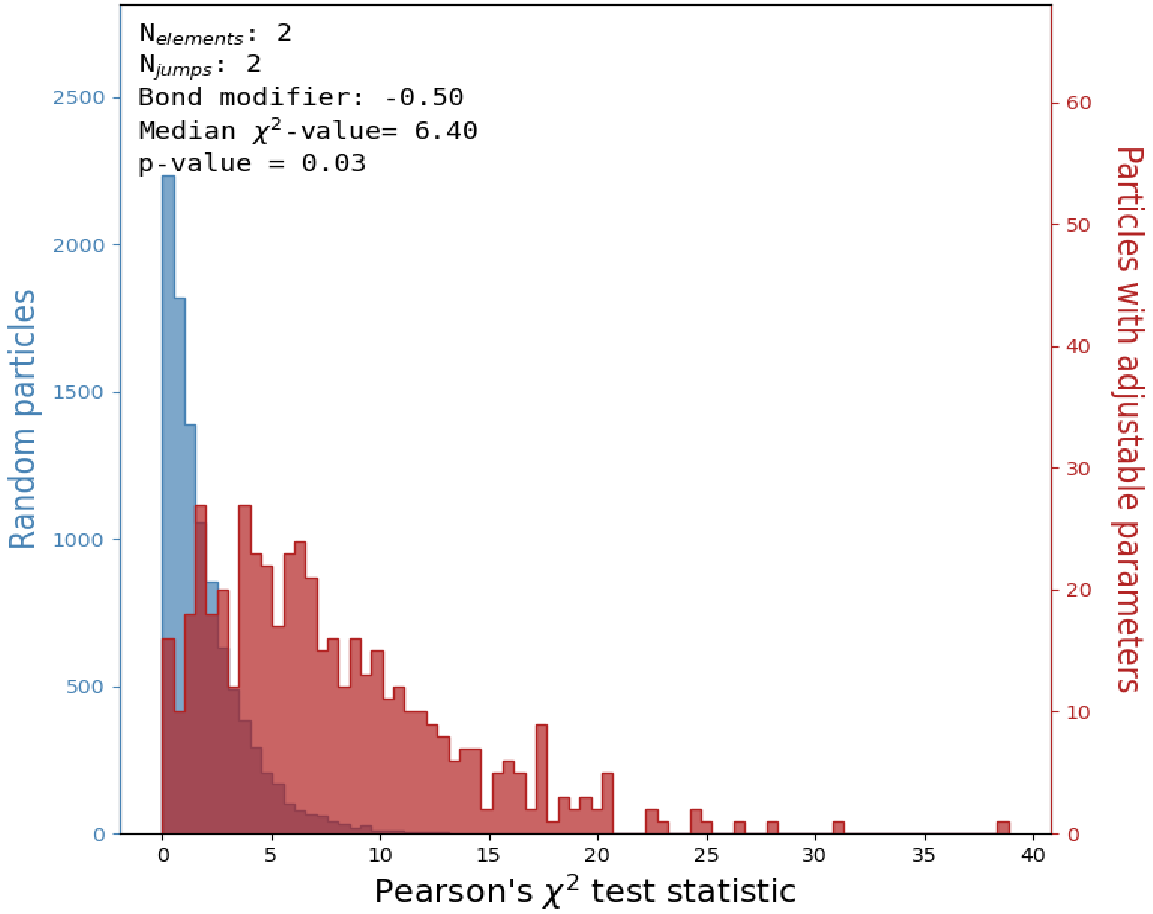

Figure S31 χ2-value distributions for 10000 randomly configured particles (0 jumps/0.00 score adjustment) in blue compared to 500 particles constructed with 2 jumps and -0.50 score for heterogenous bonds in red for a two-element particle.

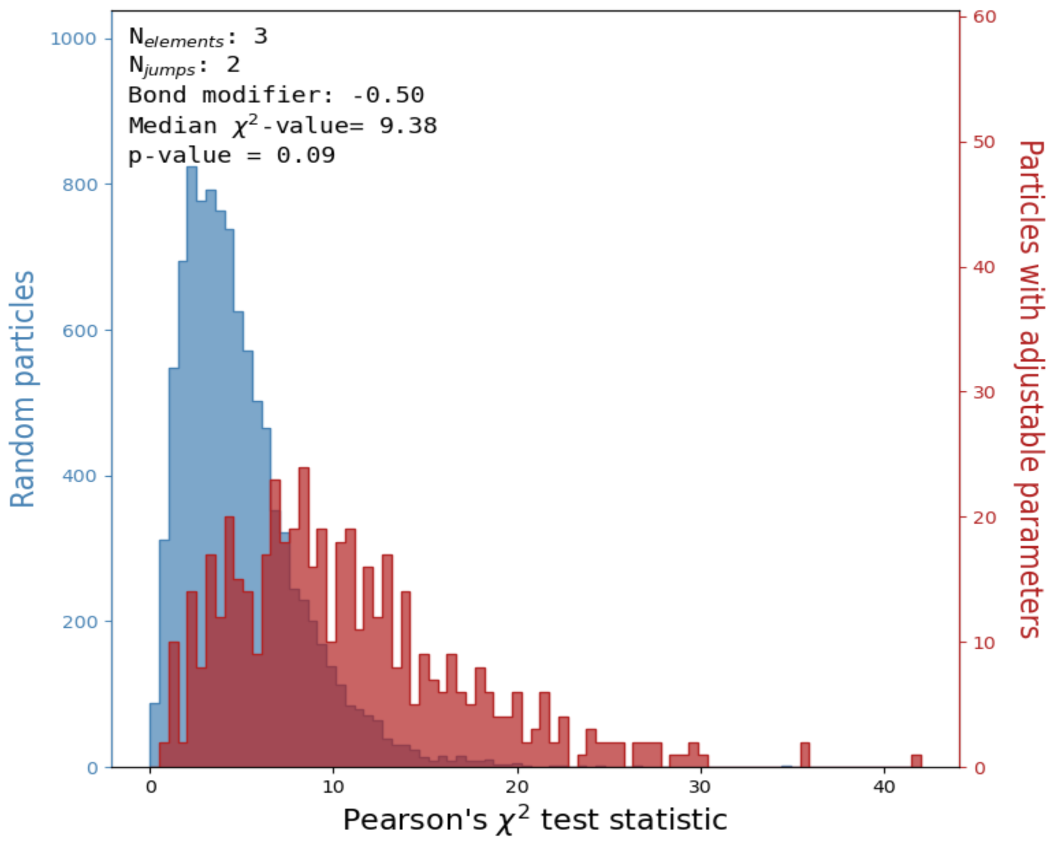

Figure S32 χ2-value distributions for 10000 randomly configured particles (0 jumps/0.00 score adjustment) in blue compared to 500 particles constructed with 2 jumps and -0.50 score for heterogenous bonds in red for a three-element particle.

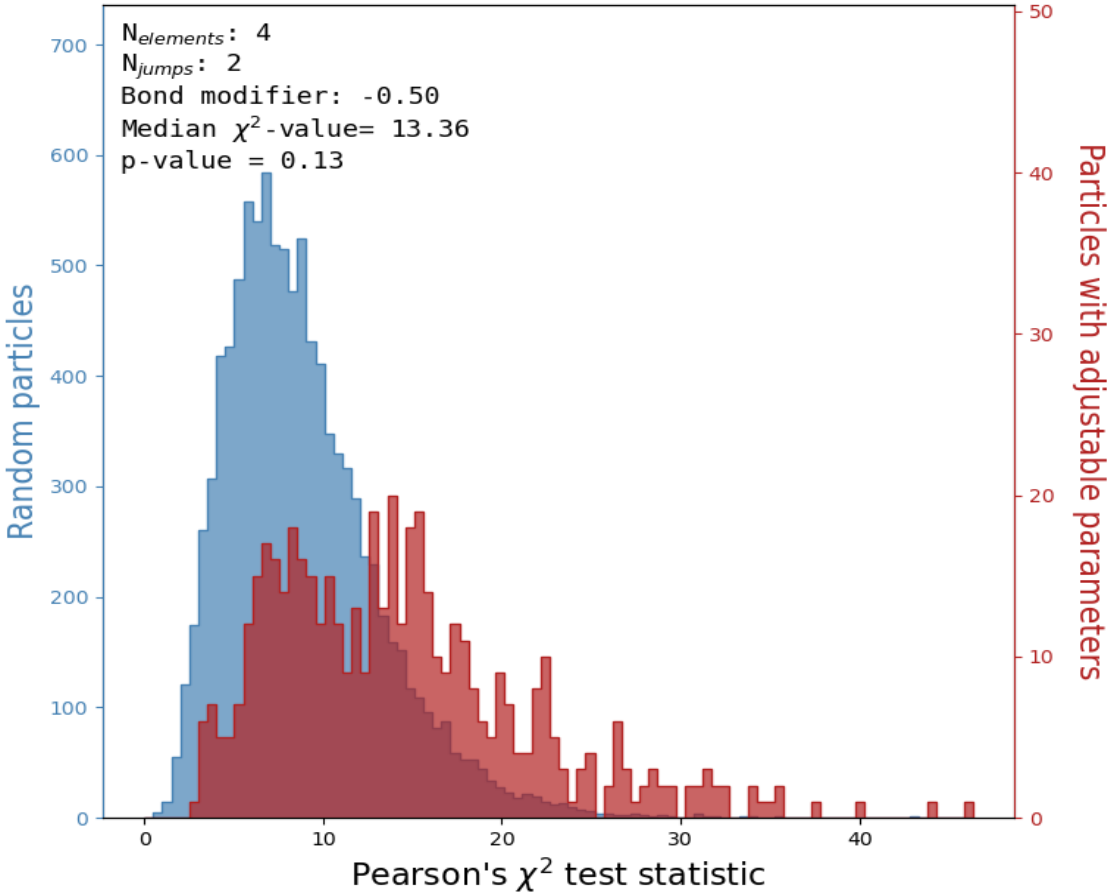

Figure S33 χ2-value distributions for 10000 randomly configured particles (0 jumps/0.00 score adjustment) in blue compared to 500 particles constructed with 2 jumps and -0.50 score for heterogenous bonds in red for a four-element particle.

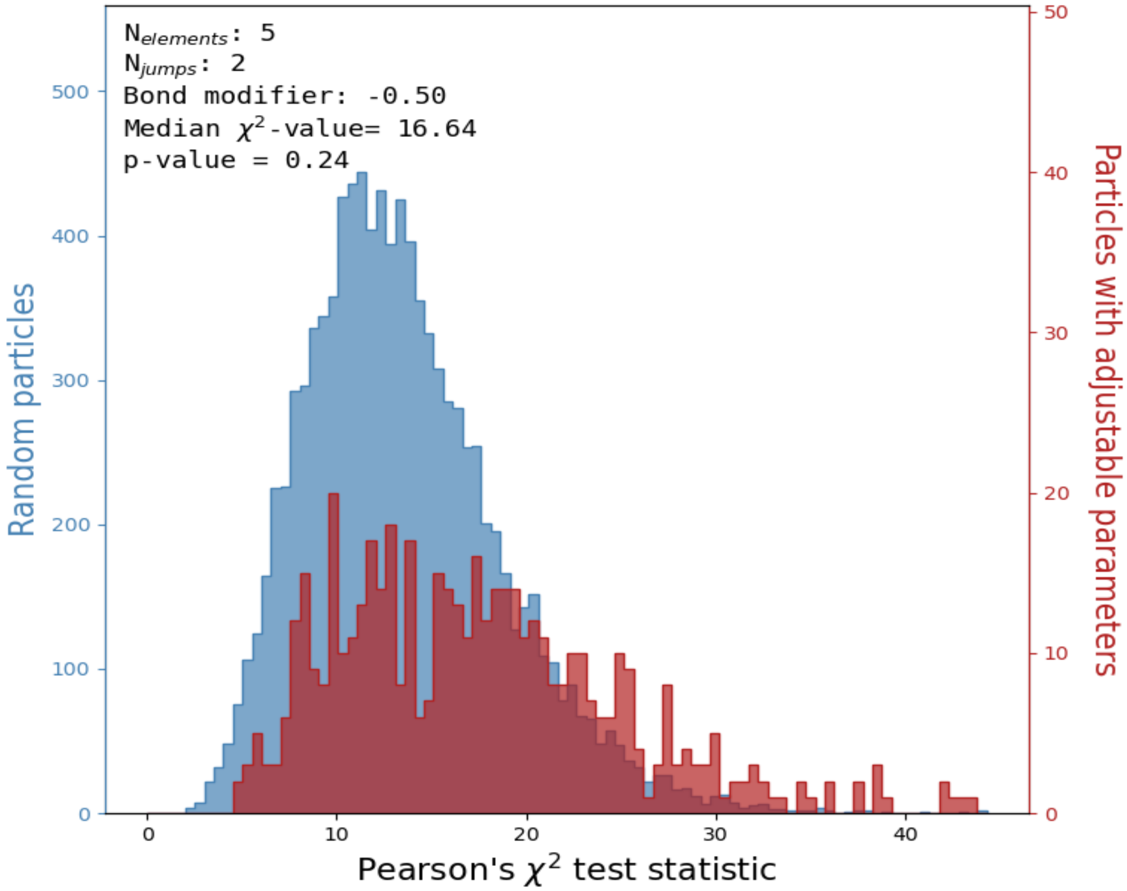

Figure S34 χ2-value distributions for 10000 randomly configured particles (0 jumps/0.00 score adjustment) in blue compared to 500 particles constructed with 2 jumps and -0.50 score for heterogenous bonds in red for a five-element particle.

Refinement tables

Results from Rietveld refinement of in situ XRD patterns of bimetallic systems (final frames, 500 °C)

Table S4 Fitted parameters for Rietveld refinement on the diffraction pattern of the last frame in the Rh-Ir bimetallic system

	R_{wp} / %	R_{exp} / %	χ^2		
	12.4	0.62	402		
fcc					
	R_{Bragg}/ %	R_F / %	Scale factor	*a* / Å	
	4.35	4.16	5.77(2)*10^{-10}	3.8496(1)	
	U	V	W	X	Y
	0.020	-0.017	0.007	0.287	0.027

Table S5 Fitted parameters for Rietveld refinement on the diffraction pattern of the last frame in the Ru-Os bimetallic system

	R_{wp} / %	R_{exp} / %	χ^2		
	9.49	0.55	298		
hcp					
	R_{Bragg} / %	R_F / %	Scale factor	*a* / Å	*c* / Å
	5.05	4.44	2.72(6)*10^{-7}	2.7383(1)	4.3394(1)
	U	V	W	X	Y
	0.652	-0.093	0.006	0.209	0.142

Table S6 Fitted parameters for Rietveld refinement on the diffraction pattern of the last frame in the Rh-Pt bimetallic system

	R_{wp} / %	R_{exp} / %	χ^2		
	19.8	0.83	568		
***fcc* - 1**					
Fraction / wt.%	R_{Bragg}/ %	R_F / %	Scale factor	*a* / Å	
74.1(4)	23.6	19.6	2.16(8)*10^{-10}	3.8862(1)	
	U	V	W	X	Y
	0.269	-0.081	0.006	0.472	0
***fcc* - 2**					
Fraction / wt.%	R_{Bragg}/ %	R_F / %	Scale factor	*a* / Å	
19.0(2)	12.3	13.5	5.32(5)*10^{-11}	3.9393(1)	
	U	V	W	X	Y
	0.242	-0.001	0.001	0.072	0
***fcc* - 3**					
Fraction / wt.%	R_{Bragg}/ %	R_F / %	Scale factor	*a* / Å	
6.9(2)	20.3	18.6	2.09(4)*10^{-11}	3.8271(1)	
	U	V	W	X	Y
	0.166	-0.001	0.001	0	0.020

Table S7 Fitted parameters for Rietveld refinement on the diffraction pattern of the last frame in the Os-Ir bimetallic system

	R_{wp} / %	R_{exp} / %	χ^2		
	21.1	1.21	305		
hcp					
Fraction / wt.%	R_{Bragg} / %	R_F / %	Scale factor	*a* / Å	*c* / Å
56.0(7)	13.6	14.1	2.69(3)*10^{-7}	2.7233(4)	4.3380(8)
	U	V	W	X	Y
	0.266	-0.026	0.004	0.300	0.145
fcc					
Fraction / wt.%	R_{Bragg}/ %	R_F / %	Scale factor	*a* / Å	
44.0(6)	10.1	9.98	3.88(5)*10^{-7}	3.8351(7)	
	U	V	W	X	Y
	0.037	-0.020	0.003	0.465	0.104

Table S8 Fitted parameters for Rietveld refinement on the diffraction pattern of the last frame in the Rh-Os bimetallic system

	R_{wp} / %	R_{exp} / %	χ^2		
	9.50	3.12	9.26		
hcp					
Fraction / wt.%	R_{Bragg} / %	R_F / %	Scale factor	*a* / Å	*c* / Å
56.7(3)	5.43	4.81	4.77(2)*10^{-8}	2.7292(1)	4.3353(3)
	U	V	W	X	Y
	0.660	-0.040	0.004	1.041	0.093
fcc					
Fraction / wt.%	R_{Bragg}/ %	R_F / %	Scale factor	*a* / Å	
43.2(2)	4.57	3.24	6.77(3)*10^{-8}	3.8147(7)	
	U	V	W	X	Y
	0.059	-0.024	0.003	0.051	0.035

Table S9 Fitted parameters for Rietveld refinement on the diffraction pattern of the last frame in the Ir-Pt bimetallic system

	R_{wp} / %	R_{exp} / %	χ^2		
	14.4	1.5	90.1		
***fcc* - 1**					
Fraction / wt.%	R_{Bragg}/ %	R_F / %	Scale factor	*a* / Å	
15.2(2)	11.9	6.00	2.27(1)*10^{-8}	3.8316(6)	
	U	V	W	X	Y
	0.019	-0.008	0.003	0.333	0.159
***fcc* - 2**					
Fraction / wt.%	R_{Bragg}/ %	R_F / %	Scale factor	*a* / Å	
64.8(10)	7.77	3.36	9.38(10)*10^{-8}	3.8748(7)	
	U	V	W	X	Y
	2.542	-0.040	0.004	0.741	0.085
***fcc* - 3**					
Fraction / wt.%	R_{Bragg}/ %	R_F / %	Scale factor	*a* / Å	
20.1(8)	3.88	1.82	2.87(9)*10^{-8}	3.9175(2)	
	U	V	W	X	Y
	0.595	-0.002	0.001	0	0.027

Table S10 Fitted parameters for Rietveld refinement on the diffraction pattern of the last frame in the Ru-Pt bimetallic system

	R_{wp} / %	R_{exp} / %	χ^2		
	13.2	0.8	293		
***fcc* - 1**					
Fraction / wt.%	R_{Bragg}/ %	R_F / %	Scale factor	*a* / Å	
82.1(5)	15.1	5.46	$1.12(4)*10^{-6}$	3.8807(1)	
	U	V	W	X	Y
	0.460	-0.021	0.002	0.605	0.067
***fcc* - 2**					
Fraction / wt.%	R_{Bragg}/ %	R_F / %	Scale factor	*a* / Å	
9.1(2)	5.9	4.29	$1.20(3)*10^{-7}$	3.9418(1)	
	U	V	W	X	Y
	0.119	-0.001	0.001	0	0.016
hcp					
Fraction / wt.%	R_{Bragg}/ %	R_F / %	Scale factor	*a* / Å	*c* / Å
8.9(2)	29.4	17.6	$2.50(5)*10^{-7}$	2.7236(3)	4.3548(6)
	U	V	W	X	Y
	0.820	-0.003	0.003	0.800	0

Table S11 Fitted parameters for Rietveld refinement on the diffraction pattern of the last frame in the Ru-Ir bimetallic system

	R_{wp} / %	R_{exp} / %	χ^2		
	25.0	1.0	637		
fcc					
Fraction / wt.%	R_{Bragg}/ %	R_F / %	Scale factor	*a* / Å	
24.8(4)	15.8	11.0	1.00(2)*10^{-6}	3.8362(4)	
	U	V	W	X	Y
	0.152	-0.009	0.002	0.001	0.249
hcp					
Fraction / wt.%	R_{Bragg}/ %	R_F / %	Scale factor	*a* / Å	*c* / Å
75.2(7)	12.2	8.9	3.06(2)*10^{-6}	2.7236(3)	4.3548(6)
	U	V	W	X	Y
	0.596	-0.147	0.014	1.413	0.036

Tables for Rietveld refinement of ex-situ HEA samples

Table S12. Fitted parameters for Rietveld refinement on the diffraction pattern of the fcc-HEA

	R_{wp} / %	R_{exp} / %	χ^2					
	13.9	0.2	4894					
***fcc*-HEA**								
	R_{Bragg} / %	R_F / %	Scale factor	*a* / Å	U	I_G	X	Y
	6.4	3.4	2.75(1)*10^{-6}	3.8324(1)	0.015	0.017	0.216	0.025

Table S13 Fitted parameters for Rietveld refinement on the diffraction pattern of the hcp-HEA using a two-phase model (hcp + fcc)

	R_{wp} / %	R_{exp} / %	χ^2						
	14.8	3.9	15						
***hcp*-HEA**									
hcp	R_{Bragg} / %	R_F / %	Scale factor	*a* / Å	*c* / Å	U	I_G	X	Y
91(1) wt.%	6.6	4.8	2.44(2)*10^{-5}	2.7183(2)	4.3317(3)	0.272	0.001	0	0.055
fcc	R_{Bragg} / %	R_F / %	Scale factor	*a* / Å		U	I_G	X	Y
9(1) wt.%	6.5	5.4	2.31(2)*10^{-5}	3.839(2)		0.978	0	0	0.170

Table S14 Fitted parameters for Rietveld refinement on the diffraction pattern of the hcp-HEA using a a single-phase model (hcp only)

	R_{wp} / %	R_{exp} / %	χ^2						
	16.8	3.9	19						
***hcp*-HEA**									
hcp	R_{Bragg} / %	R_F / %	Scale factor	a / Å	c / Å	U	I_G	X	Y
	9.1	5.8	2.65(1)*10^{-5}	2.7178(2)	4.3340(3)	0.171	0	0.001	0.060

Tables of fit parameters of PDF refinements for ex situ characterized HEA samples

Table S15 Fitted parameters for refinement on the PDF of the fcc-HEA

***fcc*-HEA**	Rw = 0.293		
Scale factor	a / Å	adp	Sp diameter / Å
0.073(1)	2.857(1)	0.009(3)	34(1)

Table S16 Fitted parameters for refinement on the PDF of the hcp-HEA (two-phase model, top and single-phase model, bottom, respectively)

***hcp*-HEA**	Rw = 0.166				
hcp	Scale factor	a / Å	c / Å	adp	Sp diameter / Å
72(4) wt.%	0.064(3)	2.724(1)	4.336(3)	0.003(3)	60(4)
Fcc	Scale factor	a / Å		adp	Sp diameter / Å
28(4) wt.%	0.026(5)	3.84		0.003	60
***hcp*-HEA**	Rw = 0.234				
hcp	Scale factor	a / Å	c / Å	adp	Sp diameter / Å
	0.071(4)	2.724 (1)	4.337(2)	0.003(3)	57(3)

References

(1) van Beek, W.; Safonova, O. V.; Wiker, G.; Emerich, H. SNBL, a Dedicated Beamline for Combined in Situ X-Ray Diffraction, X-Ray Absorption and Raman Scattering Experiments. *Phase Transit.* **2011**, *84* (8), 726–732. https://doi.org/10.1080/01411594.2010.549944.

(2) Clausen, B. S.; Steffensen, G.; Fabius, B.; Villadsen, J.; Feidenhans'l, R.; Topsøe, H. In Situ Cell for Combined XRD and On-Line Catalysis Tests: Studies of Cu-Based Water Gas Shift and Methanol Catalysts. *J. Catal.* **1991**, *132* (2), 524–535. https://doi.org/10.1016/0021-9517(91)90168-4.

(3) Hammersley, A. P. FIT2D: A Multi-Purpose Data Reduction, Analysis and Visualization Program. *J. Appl. Crystallogr.* **2016**, *49* (2), 646–652. https://doi.org/10.1107/S1600576716000455.

(4) Kieffer, J.; Karkoulis, D. PyFAI, a Versatile Library for Azimuthal Regrouping. *J. Phys. Conf. Ser.* **2013**, *425* (20), 202012. https://doi.org/10.1088/1742-6596/425/20/202012.

(5) Prescher, C.; Prakapenka, V. B. DIOPTAS: A Program for Reduction of Two-Dimensional X-Ray Diffraction Data and Data Exploration: High Pressure Research: Vol 35, No 3. *High Press. Res.* **2015**, *35* (3), 223–230.

(6) Dyadkin, V.; Pattison, P.; Dmitriev, V.; Chernyshov, D. A New Multipurpose Diffractometer PILATUS@SNBL. *J. Synchrotron Radiat.* **2016**, *23* (3), 825–829. https://doi.org/10.1107/S1600577516002411.

(7) Rodríguez-Carvajal, J. Recent Advances in Magnetic Structure Determination by Neutron Powder Diffraction. *Phys. B Condens. Matter* **1993**, *192* (1), 55–69. https://doi.org/10.1016/0921-4526(93)90108-I.

(8) Thompson, P.; Cox, D. E.; Hastings, J. B. Rietveld Refinement of Debye–Scherrer Synchrotron X-Ray Data from Al2O3. *J. Appl. Crystallogr.* **1987**, *20* (2), 79–83. https://doi.org/10.1107/S0021889887087090.

(9) Rodríquez-Carvajal, J.; Roisnel, T. Line Broadening Analysis Using FullProf*: Determination of Microstructural Properties. *Mater. Sci. Forum* **2004**, *443–444*, 123–126. https://doi.org/10.4028/www.scientific.net/MSF.443-444.123.

(10) Bhakar, A.; Pandey, A. H.; Singh, M. N.; Upadhyay, A.; Sinha, A. K.; Gupta, S. M.; Ganguli, T.; Rai, S. K. Effect of Processing Parameters on Microstructural Properties of Lead Magnesium Niobates. *Acta Crystallogr. Sect. B Struct. Sci. Cryst. Eng. Mater.* **2017**, *73* (Pt 6), 1095–1104. https://doi.org/10.1107/S2052520617012872.

(11) Juhás, P.; Davis, T.; Farrow, C. L.; Billinge, S. J. L. PDFgetX3: A Rapid and Highly Automatable Program for Processing Powder Diffraction Data into Total Scattering Pair Distribution Functions. *J. Appl. Crystallogr.* **2013**, *46* (2), 560–566. https://doi.org/10.1107/S0021889813005190.

(12) Farrow, C. L.; Juhas, P.; Liu, J. W.; Bryndin, D.; Božin, E. S.; Bloch, J.; Proffen, T.; Billinge, S. J. L. PDFfit2 and PDFgui: Computer Programs for Studying Nanostructure in Crystals. *J. Phys. Condens. Matter* **2007**, *19* (33), 335219. https://doi.org/10.1088/0953-8984/19/33/335219.

(13) Ravel, B.; Newville, M. ATHENA, ARTEMIS, HEPHAESTUS: Data Analysis for X-Ray Absorption Spectroscopy Using IFEFFIT. *J. Synchrotron Radiat.* **2005**, *12* (4), 537–541. https://doi.org/10.1107/S0909049505012719.

(14) Newville, M. Larch: An Analysis Package for XAFS and Related Spectroscopies. *J. Phys. Conf. Ser.* **2013**, *430* (1), 012007. https://doi.org/10.1088/1742-6596/430/1/012007.
(15) Yusenko, K. V.; Gromilov, S. A.; Baidina, I. A.; Shubin, Yu. V.; Korol'kov, I. V.; Drebushchak, T. N.; Basova, T. V.; Korenev, S. V. Synthesis, Structure, and Thermal Decomposition of Chloropentamminerhodium(III) Hexabromoplatinate(IV). *J. Struct. Chem.* **2002**, *43* (4), 649–655. https://doi.org/10.1023/A:1022004721745.
(16) Yusenko, K. V.; Riva, S.; Carvalho, P. A.; Yusenko, M. V.; Arnaboldi, S.; Sukhikh, A. S.; Hanfland, M.; Gromilov, S. A. First Hexagonal Close Packed High-Entropy Alloy with Outstanding Stability under Extreme Conditions and Electrocatalytic Activity for Methanol Oxidation. *Scr. Mater.* **2017**, *138*, 22–27. https://doi.org/10.1016/j.scriptamat.2017.05.022.
(17) Kittel, C. *Introduction to Solid State Physics*, 8th ed.; Wiley: New York, 2004.
(18) Aqra, F.; Ayyad, A. Surface Energies of Metals in Both Liquid and Solid States. *Appl. Surf. Sci.* **2011**, *257* (15), 6372–6379. https://doi.org/10.1016/j.apsusc.2011.01.123.
(19) *CRC Handbook of Chemistry and Physics*, 95th ed.; Haynes, W. M., Ed.; CRC Press, Taylor & Francis Group: Boca Raton, FL, 2014.
(20) Schmidt, T. J.; Gasteiger, H. A.; Stäb, G. D.; Urban, P. M.; Kolb, D. M.; Behm, R. J. Characterization of High-Surface-Area Electrocatalysts Using a Rotating Disk Electrode Configuration. *J. Electrochem. Soc.* **1998**, *145* (7), 2354. https://doi.org/10.1149/1.1838642.
(21) Arblaster, J. W. Crystallographic Properties of Iridium. *Platin. Met. Rev* **2010**, *54* (2), 93.
(22) Arblaster, J. W. Crystallographic Properties of Osmium. *Platin. Met. Rev* **2013**, *57* (3), 177.
(23) Arblaster, J. W. Crystallographic Properties of Rhodium. *Platin. Met. Rev* **1997**, *41* (4), 184.
(24) Arblaster, J. W. Crystallographic Properties of Platinum. *Platin. Met. Rev.* **2006**, *50* (3), 118–119. https://doi.org/10.1595/147106706X129088.
(25) Arblaster, J. W. Crystallographic Properties of Ruthenium. *Platin. Met. Rev* **2013**, *57* (2), 127.
(26) Pedregosa, F.; Varoquaux, G.; Gramfort, A.; Michel, V.; Thirion, B.; Grisel, O.; Blondel, M.; Prettenhofer, P.; Weiss, R.; Dubourg, V.; Vanderplas, J.; Passos, A.; Cournapeau, D.; Brucher, M.; Perrot, M.; Duchesnay, E. Scikit-Learn: Machine Learning in Python. *J. Mach. Learn. Res.* **2011**.
